# Sustainable Biodiesel Production over a Novel Magnetic Solid Acid Catalyst Derived from Copper Ferrite: Statistical Optimization and Catalytic Performance Evaluation

**DOI:** 10.1021/acsomega.6c08033

**Published:** 2026-09-17

**Authors:** Gustavo Ferreira Leal, Matheus Arrais Gonçalves, Hiarla Cristina Lima dos Santos, Alexandre da Cas Viegas, Erica Karine Lourenço Mares, Leyvison Rafael Vieira da Conceição

**Affiliations:** † 37871 Federal University of Pará, Institute of Exact and Natural Sciences, Graduate in Chemistry Program, Laboratory of Catalysis and Oleochemical, Belém, Pará 66075−110, Brazil; ‡ 28117 Federal University of Rio Grande do Sul, Institute of Physics, Porto Alegre, Rio Grande do Sul 90035−190, Brazil; § Federal Institute of Education, Science and Technology of Pará, Department of Education, Science and Teacher Education, Paragominas, Pará 68629−020, Brazil

## Abstract

In this study, a novel magnetic solid acid catalyst based on molybdenum oxide-impregnated copper ferrite was synthesized and applied to methyl transesterification from waste cooking oil (WCO). The catalytic solid was characterized by surface acidity, XRD, FTIR, SEM, EDS, and VSM techniques. A 2^4^ face-centered central composite design (FCCD) and a linear regression mathematical model were developed to describe the behavior of the ester content of the biodiesel produced as a function of the independent reaction variables: temperature (120–180 °C); MeOH:WCO molar ratio (10:1–50:1); time (1–5 h); and catalyst concentration (2–10 wt %). The optimized experimental results yielded a biodiesel with 95.6% ester content under the following optimized reaction conditions: temperature of 155 °C, MeOH:WCO molar ratio of 35:1, catalyst concentration of 8 wt %, and reaction time of 3.5 h. The developed mathematical model exhibited a relative error of less than 5% between predicted and observed values, demonstrating its robustness. Furthermore, the catalyst exhibited excellent catalytic stability over four reaction cycles, with ester conversion remaining above 85%, indicating great reusability potential.

## Graphical abstract

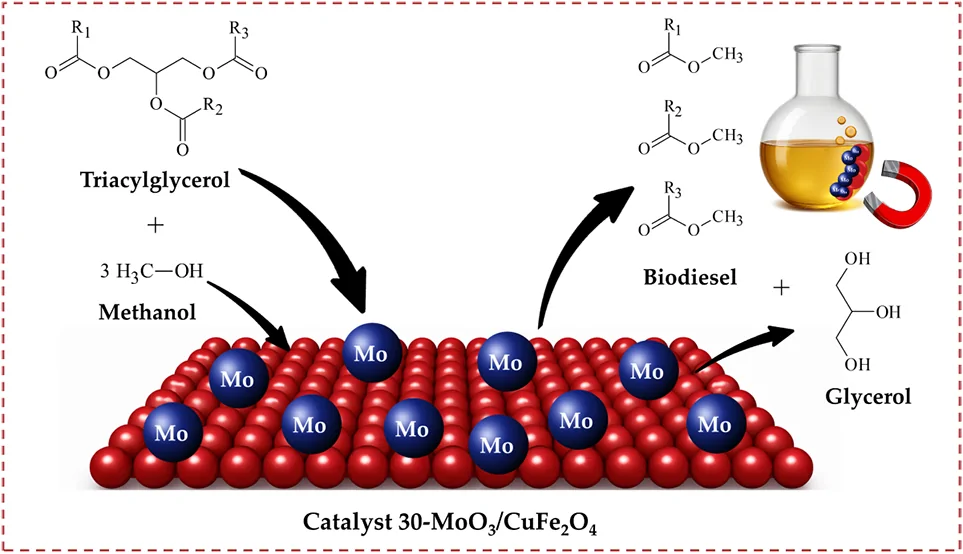

## 1 Introduction

The consumption of fossil fuels has grown exponentially due to population growth and increased consumer demand, leading to the depletion of nonrenewable resources and an unprecedented environmental crisis.^1,2^ Many fossil fuels, including gasoline, coal, and petroleum, have diverse applications across various economic sectors; however, they are responsible for numerous impacts on nature and the environment.^3,4^ Consequently, there is a vital need for renewable energy sources (e.g., ethanol, biodiesel, hydrogen, etc.) to supplement the already established fossil fuel sources, making it imperative for numerous researchers to conduct diverse studies in this area.^5,6^

The use of renewable raw materials derived from reuse, such as waste cooking oil (WCO), will not only reduce production costs but also prevent potential environmental contamination from improper disposal in sewage systems.^7–9^ Among renewable energy sources, biodiesel has emerged as the most prominent, which is attributed to several factors, including its biodegradability, nontoxicity, origin from the reuse of other materials, and low emission of sulfur and nitrogen oxides, among others.^8^ Moreover, biodiesel exhibits physicochemical properties that closely resemble those of fossil fuels, enabling its use as an alternative fuel. It is produced from vegetable oils and animal fats through catalytic esterification or transesterification of long-chain fatty acids and triglycerides.^9^ Commonly, a large portion of biodiesel synthesis costs is attributed to obtaining the oil used in the reactions, potentially accounting for up to 65% of expenses. Therefore, due to the very low cost of WCO compared to other oil sources, its use can have a considerable impact on reducing the cost of biodiesel synthesis.^10,11^

Moreover, the high residual acidity value in the WCO feedstock makes it ideal for transesterification reactions with acid catalysts, as the use of basic catalysts is precluded by the occurrence of basic hydrolysis, also known as saponification, instead of biodiesel formation.^2,9^ Although a two-step process involving homogeneous acid esterification followed by base-catalyzed transesterification is a viable strategy, it requires additional reagents, generates waste, and complicates catalyst recovery. In this context, solid acid catalysts offer a simplified route, as they can promote both esterification and transesterification in a single step while also enabling easier catalyst separation and reuse. The conversion of WCO into biodiesel can involve the use of homogeneous, heterogeneous, or enzymatic catalysts, as well as different approaches in esterification/transesterification reactions.^12,13^ The use of heterogeneous catalysts proves to be an excellent alternative to both syntheses with homogeneous catalysts and enzymatic processes, due to the reduced effort and resources required for biodiesel purification, as well as the easy recovery and reuse of the catalyst and the less intensive use of equipment in biodiesel production.^12^

In this context, the foremost examples of heterogeneous catalysis include zeolites, mesoporous silicas, carbons, and metal oxides.^14–17^ Furthermore, it can be highlighted that among heterogeneous catalysts, those with acidic properties offer several advantages over basic heterogeneous catalysts, such as the impossibility of basic hydrolysis occurring when also using WCO.^15,16^ Thus, among metal oxide catalysts, which are prominently featured in the literature, molybdenum oxide (MoO_3_) stands out due to its strong Lewis acidity and excellent catalytic capacity in biodiesel production, in addition to possessing high versatility in redox properties and a very stable oxidation state (Mo^6+^).^17–19^

Among heterogeneous catalysts, those with magnetic properties are increasingly attracting attention as they facilitate catalyst recovery after biodiesel synthesis. They can be separated by centrifugation, filtration, or applying an external magnetic field, thus saving operational costs and reducing waste generation by enabling catalyst reuse over multiple reaction cycles.^20,21^ Various materials exhibit such magnetic properties, including hematite (Fe_2_O_3_) and magnetite (Fe_3_O_4_), with special emphasis on ferrites (MFe_2_O_4_), where M can be numerous transition metals, making them highly versatile and adaptable.^22–24^

Recent studies, such as that by Tamoradi et al.,^25^ evaluate the impregnation of magnesium oxide (MgO) combined with graphene oxide (GO) and copper ferrite (CuFe_2_O_4_) for biodiesel synthesis from waste corn oil and methanol. The biodiesel obtained achieved 93.55% ester conversion under the following optimal reaction conditions: temperature of 65 °C, MeOH:WCO molar ratio of 13:1, reaction time of 7 h, and catalyst concentration of 8%; it should be noted that the catalyst in question has a basic active phase. Furthermore, the study by Brito et al.^26^ shows the impregnation of MoO_3_ onto niobium pentoxide (Nb_2_O_5_) also for methyl biodiesel synthesis from WCO. The optimal reaction conditions obtained were as follows: temperature of 145 °C, MeOH:WCO molar ratio of 20:1, reaction time of 2.5 h, and catalyst concentration of 6 wt %, yielding 94.2% ester conversion in the catalytic tests.

The present study aims at the application and optimization of a heterogeneous magnetic acid catalyst, composed of MoO_3_ impregnated on CuFe_2_O_4_, using waste cooking oil and methanol for biodiesel production. It should also be noted that this is the first study to evaluate the interaction between molybdenum and nickel ferrite in a transesterification process. Furthermore, the effects of the variables involved in the transesterification reaction, such as reaction temperature, MeOH:WCO molar ratio, reaction time, and catalyst concentration, were investigated through response surface methodology (RSM) and the development of a linear regression mathematical model.

## 2 Materials and Methods

### 2.1 Materials

All reagents used were of analytical grade. Copper­(II) acetate (CuC_4_H_6_O_4_·H_2_O, 98%) purchased from Dinâmica (Brazil), iron­(III) nitrate (Fe­(NO_3_)_3_·9H_2_O, 98%) purchased from Neon (Brazil), and ammonium heptamolybdate ((NH_4_)_6_Mo_7_O_24_·4H_2_O, 99%) purchased from Dinâmica (Brazil) were all used in the preparation of the catalyst. Sodium hydroxide (NaOH, 97%) and hydrochloric acid (HCl, 37%) were purchased from Neon (Brazil) and Isofar (Brazil), respectively, and were used to determine the catalyst’s surface acidity. Methyl alcohol (CH_3_OH, 99.8%) purchased from Dinâmica (Brazil) and waste cooking oil (WCO) with an acid value of 4.5 mg KOH g^–1^ (corresponding to approximately 2.3 wt % free fatty acids), collected from a restaurant at the Federal University of Pará, were used in the transesterification reaction. Methyl heptadecanoate (C_18_H_36_O_2_, 99%) purchased from Sigma-Aldrich (USA) and heptane (C_7_H_16_, 99.5%) were used for gas chromatographic analysis. Ethanol (C_2_H_5_OH, 99.8%) purchased from Êxodo Científica (Brazil) and hexane (C_6_H_14_, 99.5%) purchased from Dinâmica (Brazil) were used in the catalyst washing process for subsequent reuse.

### 2.2 Magnetic Solid Catalyst Synthesis (MoO_3_/CuFe_2_O_4_)

#### 2.2.1 Synthesis of Copper Ferrite

Figure 1 (I) illustrates the synthesis of copper ferrite (CuFe_2_O_4_). Initially, the ferrite, used as the catalyst support, was prepared by the coprecipitation method according to an adapted methodology from Gonçalves et al.^27^ For this purpose, 8.08 g of Fe­(NO_3_)_3_·9H_2_O was dissolved in 200 mL of distilled water, followed by the addition of 5 mL of HCl. Subsequently, mechanical stirring was applied, and 2 g of CuC_4_H_6_O_4_·H_2_O was added at a 1:2 molar ratio at room temperature for 10 min. Then, a 4 mol L^–1^ NaOH solution was added dropwise to adjust the pH of the mixture to 12. Upon completion of the precipitation process, the system was maintained under mechanical stirring for 4 h with heating to 65 °C. Subsequently, the product obtained was washed with distilled water until the filtrate reached neutrality (pH = 7). Finally, the material was dried in an oven at 100 °C for 12 h and calcined at 500 °C for 3 h (at a heating rate of 5 °C min^–1^) to obtain CuFe_2_O_4_.

**Figure 1:**
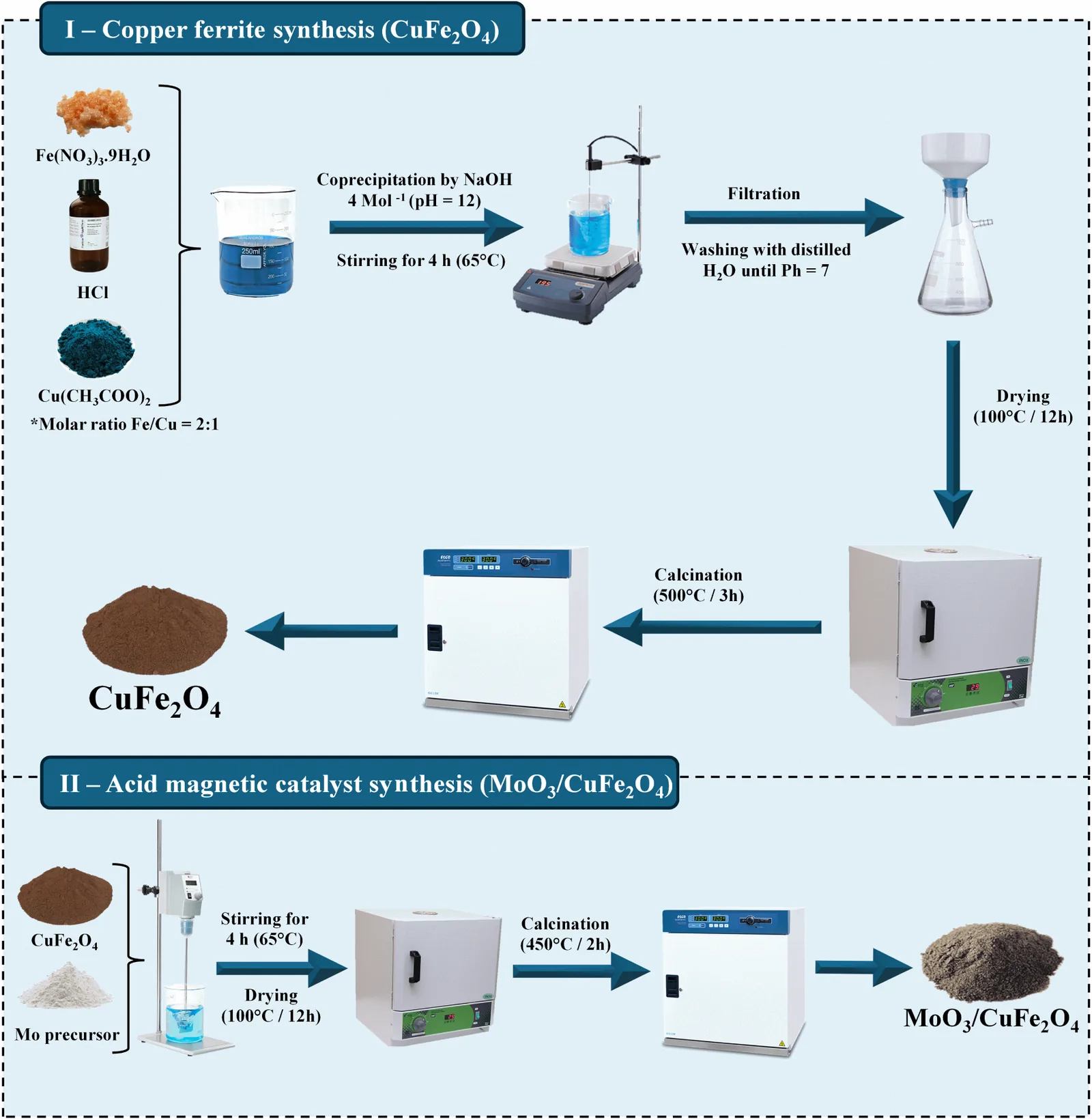
Schematic diagram of (I) CuFe_2_O_4_ and (II) MoO_3_/CuFe_2_O_4_ synthesis.

#### 2.2.2 Loading of the Active Phase onto the Support

The impregnation step of the active phase onto the support was carried out according to the methodology described by Santos et al.^28^ with adaptations, as shown in Figure 1 (II). A series of catalysts were synthesized considering different molybdenum metal loadings in the active phase, ranging from 10% to 40%. Thus, in a typical procedure, a mass of 1.0 g of CuFe_2_O_4_ was weighed and added to a beaker along with 40 mL of distilled water, followed by the addition of 0.78 g of (NH_4_)_6_Mo_7_O_24_·4H_2_O, considering an impregnation concentration of 30% molybdenum active phase. This suspension was maintained under constant mechanical stirring for 2 h at room temperature. Subsequently, the mixture was dried in an oven at 100 °C for 10 h, followed by the calcination step at 450 °C for 2 h (at a heating rate of 10 °C/min), in order to obtain the β-MoO_3_/CuFe_2_O_4_ catalyst, where β corresponds to the molybdenum metal loading impregnated onto the CuFe_2_O_4_ support.

### 2.3 Techniques for Material Characterization

The characterization of MoO_3_, CuFe_2_O_4_, and the MoO_3_/CuFe_2_O_4_ catalyst was performed using several techniques. Surface acidity was determined by placing 0.1 g of sample in 20 mL of standardized 0.1 mol L^–1^ NaOH solution, which remained under stirring for at least 24 h at room temperature. Subsequently, the sample was centrifuged, and the supernatant was titrated with a standardized 0.1 mol L^–1^ HCl solution, using phenolphthalein as an indicator.^29^ Surface acidity was calculated based on eq 1:

1 Surface Acidity={[(Mb⁡×⁡VbMa)−Va]msample}⁡×⁡Ma

where M_b_ is the NaOH molarity; M_a_ is the HCl molarity; V_a_ and V_b_ are the volumes of HCl and NaOH used, respectively; and m_sample_ is the sample mass.

X-ray diffraction (XRD) patterns of MoO_3_, CuFe_2_O_4_, and MoO_3_/CuFe_2_O_4_ were obtained using a PANalytical EMPYREAN diffractometer with Cu (Kα = 1.541874 Å) radiation, operating at 40 kV and 30 mA, over a 2θ range of 8° to 70°. The Fourier Transform Infrared (FTIR) spectra of MoO_3_, CuFe_2_O_4_, and MoO_3_/CuFe_2_O_4_ were recorded on a PerkinElmer FTIR System Spectrum GX spectrometer in the range of 2000–400 cm^–1^ with a resolution of 4 cm^–1^ and a total of 32 scans. The surface morphology of MoO_3_, CuFe_2_O_4_, and MoO_3_/CuFe_2_O_4_ was determined by Scanning Electron Microscopy (SEM) using a Tescan VEGA 3 LMU microscope. Surface elemental analyses of CuFe_2_O_4_ and MoO_3_/CuFe_2_O_4_ were performed by Energy-Dispersive X-ray Spectroscopy (EDS) using an Oxford microanalysis system, AZTec Energy X-Act model, with a resolution of 129 eV. The magnetic properties of CuFe_2_O_4_ and MoO_3_/CuFe_2_O_4_ were analyzed by Vibrating Sample Magnetometry (VSM) using a Microsense EZ9 magnetometer at room temperature, under an applied magnetic field ranging from −20,000 to 20,000 Oersted (Oe).

### 2.4 Transesterification Process

A PARR 5000 Multireactor was used to carry out all transesterification reactions. Stirring was set at 900 rpm, with variation of the following reaction conditions: temperature (120–180 °C); MeOH:WCO molar ratio (10:1–50:1); reaction time (1–5 h); and catalyst concentration (2–10 wt %), expressed relative to the mass of WCO, using 12 g of WCO. Upon completion of the reaction, the magnetic catalyst was separated from the reaction mixture using an external magnetic field. The reaction products were subsequently transferred to a separatory funnel for phase separation, followed by washing of the biodiesel phase with 500 mL of distilled water at 80 °C to eliminate residual glycerol. The purified biodiesel was then dried at 50 °C for 12 h and stored for subsequent gas chromatography (GC) analysis.

### 2.5 Statistical Analysis and Response Surface Methodology (RSM)

A 2^4^ face-centered central composite design (FCCD) was developed in order to evaluate the impact of the independent variables of the transesterification reaction on the ester content of the biodiesel produced. Reaction temperature, MeOH:WCO molar ratio, catalyst loading, and reaction time were selected as the independent variables and designated as X_1_ (120–180 °C), X_2_ (10:1–50:1), X_3_ (2–10 wt %), and X_4_ (2–5 h), respectively. Ester content (Y_1_) was adopted as the response variable. The selected ranges for the independent variables were defined based on previous literature on acid-catalyzed transesterification of WCO using ferrite-based catalysts, as well as on preliminary screening experiments conducted in our laboratory. The experimental matrix consisted of 30 experiments, comprising 16 factorial points (−1 and +1), 8 axial points, and 6 center points (0), as summarized in Table 1.

**Table 1:** Independent Variables and Coded Levels in the Experimental Design

			**Range and level**		
Independent variables	Unit	Code	–1	0	+1
Reaction temperature	°C	X_1_	120	150	180
MeOH:oil molar ratio	mol/mol	X_2_	10	30	50
Catalyst concentration	wt %	X_3_	2	6	10
Reaction time	h	X_4_	1	3	5

Furthermore, Response Surface Methodology (RSM) was applied to investigate the influence of the process variables and identify the optimal conditions for biodiesel production. A polynomial regression model (eq 2) was subsequently fitted to describe the relationship between the response variable, ester content (Y_1_), and the independent variables: reaction temperature (X_1_), MeOH:WCO molar ratio (X_2_), catalyst loading (X_3_), and reaction time (X_4_).

2 $$Y_{1}=\alpha_{0}+\overset{k}{\underset{i=1}{\sum }}\alpha_{i}X_{i}+\overset{k}{\underset{i=1}{\sum }}\alpha_{\mathrm{ii}}X_{i}^{2}+\overset{k}{\underset{i<1}{\sum }}\alpha_{\mathrm{ij}}X_{i}X_{j}+e$$

where Y_1_ denotes the response variable; *α*_0_ is the intercept at the center point; *α*_i_ corresponds to the first-order linear coefficients; *α*_ij_ corresponds to the interaction coefficients; *α*_ii_ represents the second-order (quadratic) coefficients; and e denotes the random experimental error.

The significance of the quadratic polynomial model was assessed by analysis of variance (ANOVA) with a 95% confidence level. In addition, Fisher’s F-test was employed to evaluate the adequacy of the proposed model. The F-statistic was calculated using eq 3, and all statistical analyses were performed with TIBCO Statistica software (version 14).

3 $$F-\mathrm{value}=\frac{{\mathrm{MS}}_{\mathrm{SSR}}}{{\mathrm{MS}}_{\mathrm{SSE}}}$$

where MS_SSR_ is the mean square of regression and MS_SSE_ is the mean square of residual.

### 2.6 Physicochemical Characterization of Biodiesel

The ester content of the biodiesel samples produced by transesterification was determined by gas chromatography following an adapted methodology based on the European standard EN 14103, as described by Gonçalves et al.^21^ The ester content determined by GC (EN 14103) directly corresponds to the conversion of triglycerides into methyl esters and is therefore used throughout this work as a measure of reaction efficiency. A Varian CP 3800 gas chromatograph was used, equipped with a Flame Ionization Detector (FID) and a CP WAX 52 CB capillary column (30 m in length, 0.32 mm in diameter, and 0.25 μm film thickness). Methyl heptadecanoate was used as the internal standard and heptane as the solvent. Furthermore, the oven temperature program was set from 170 to 250 °C (the same temperature as the FID) at a heating rate of 10 °C min^–1^. Helium was used as the carrier gas at a flow rate of 1.0 mL min^–1^, and the injection volume was 1 μL of sample. The ester content (Y_1_) was calculated using eq 4:

4 $$Y_{1}\left(\%\right)=\frac{\left(\sum A_{T}\right)-A_{\mathrm{IS}}}{A_{\mathrm{IS}}}\times \frac{C_{\mathrm{IS}}}{C_{B100}}\times 100$$

where ∑A_T_ is the total peak area from methyl ester peaks; A_IS_ is the peak area of the internal standard (methyl heptadecanoate); C_IS_ is the concentration of the internal standard solution (mg mL^–1^); and C_B100_ is the concentration of the biodiesel solution after dilution (mg L^–1^).

### 2.7 Catalyst Reuse Study

Catalyst reusability was investigated under the optimized transesterification conditions. At the end of each reaction cycle, the magnetic catalyst was separated from the reaction mixture by applying an external magnetic field, followed by two washing steps with hexane (10 mL each) and one washing step with ethanol (10 mL). The recovered catalyst was then dried at 100 °C for 10 h and reused in subsequent transesterification reactions.

## 3 Results and Discussion

### 3.1 Effect of Molybdenum Content on Catalyst Composition

The influence of molybdenum concentration at loadings of 10, 20, 30, and 40% in the final catalyst composition was evaluated with respect to the biodiesel obtained, as shown in Figure 2. It is worth noting that these loadings were chosen based on previous literature on supported ferrites and on internal screening experiments, which indicated that values below 10% provided insufficient acidity, while values above 40% led to agglomeration and reduced catalytic performance. The reactions were carried out under the following nonoptimized conditions: reaction temperature of 150 °C, MeOH:WCO molar ratio of 30:1, catalyst concentration of 6 wt %, and reaction time of 3 h.

**Figure 2:**
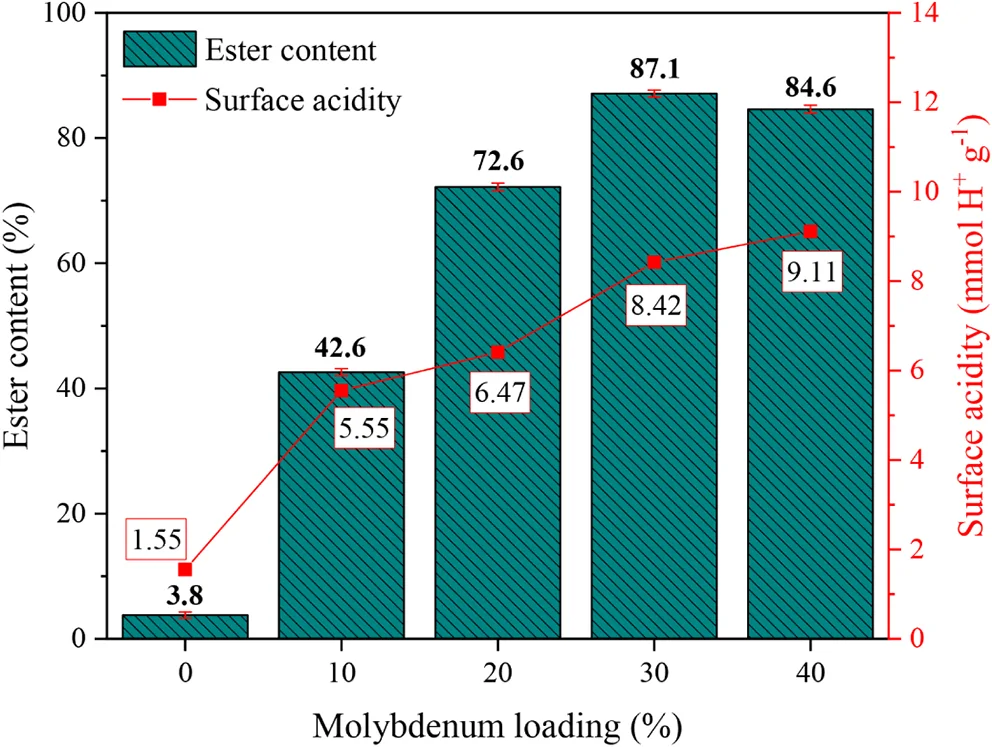
Influence of molybdenum content.

The ester content of the biodiesels for catalysts containing 10, 20, 30, and 40% molybdenum was 42.6% (±0.578), 72.2% (±0.593), 87.1% (±0.549), and 84.6% (±0.633), respectively. All experiments were performed in triplicate; the reported values represent the mean ± standard error of the mean. Thus, it can be observed that increasing the molybdenum concentration in the catalyst leads to an increase in the ester content of the biodiesels. This behavior is due to the increase in the number of acid sites on the catalyst surface, which results in enhanced reaction conversion because of ionic exchange on the surface during the triglyceride conversion process to esters.^30,31^ Consequently, a progressive increase in surface acidity was observed with increasing molybdenum loading, reflecting the greater concentration of Lewis acid sites provided by the impregnated MoO_3_.^31–33^ The catalysts containing 10, 20, 30, and 40 wt % molybdenum exhibited surface acidity values of 5.55, 6.47, 8.42, and 9.11 mmol H^+^ g^–1^, respectively.

Furthermore, the CuFe_2_O_4_ support without molybdenum impregnation (0 wt % Mo) exhibited a surface acidity of 1.55 mmol H^+^ g^–1^ and an ester conversion of only 3.8% (±0.493), indicating that the magnetic support alone does not provide a sufficient density of acid sites to effectively catalyze the transesterification reaction. Increasing the molybdenum loading enhanced the surface acidity, reaching a maximum value of 9.11 mmol H^+^ g^–1^ at 40 wt % Mo.

However, under these conditions, the ester content decreased slightly to 84.6% (±0.633). As shown in Figure 2, the error bars corresponding to the 30 and 40 wt % Mo loadings do not overlap, confirming that this decrease is statistically significant. This behavior suggests that excessive molybdenum loading promotes the formation of MoO_3_ agglomerates during the impregnation process, which reduces the available surface area by blocking the support pores and limiting the accessibility of the reactants to the active sites. Therefore, the 30-MoO_3_/CuFe_2_O_4_ catalyst, containing 30 wt % impregnated molybdenum, was selected for the subsequent transesterification studies.

### 3.2 Characterization of the Magnetic Catalyst 30-MoO_3_/CuFe_2_O_4_

The X-ray diffraction patterns of MoO_3_, CuFe_2_O_4_, and 30-MoO_3_/CuFe_2_O_4_ are shown in Figure 3. The diffractogram corresponding to MoO_3_ reveals the presence of well-defined characteristic peaks of both the hexagonal structure (h-MoO_3_) and the orthorhombic polymorphic structures (α-MoO_3_). Thus, the main peaks referring to h-MoO_3_ are observed at 25.7°, 29.6°, 33.8°, 45.8°, and 46.2°, which were directly indexed to the crystallographic planes (210), (300), (220), (410), and (404), respectively. Similarly, the peaks corresponding to the α-MoO_3_ structure appear at 12.8°, 23.2°, 27.3°, 33.1°, 35.4°, 38.8°, 49.3°, 55.2°, and 58.8°, with the crystallographic planes (020), (110), (021), (101), (041), (060), (002), (112), and (081), respectively, according to the standard cards (JCPDS: 00–005–0508 and JCPDS: 00–021–0569) for this material.^32,33^

**Figure 3:**
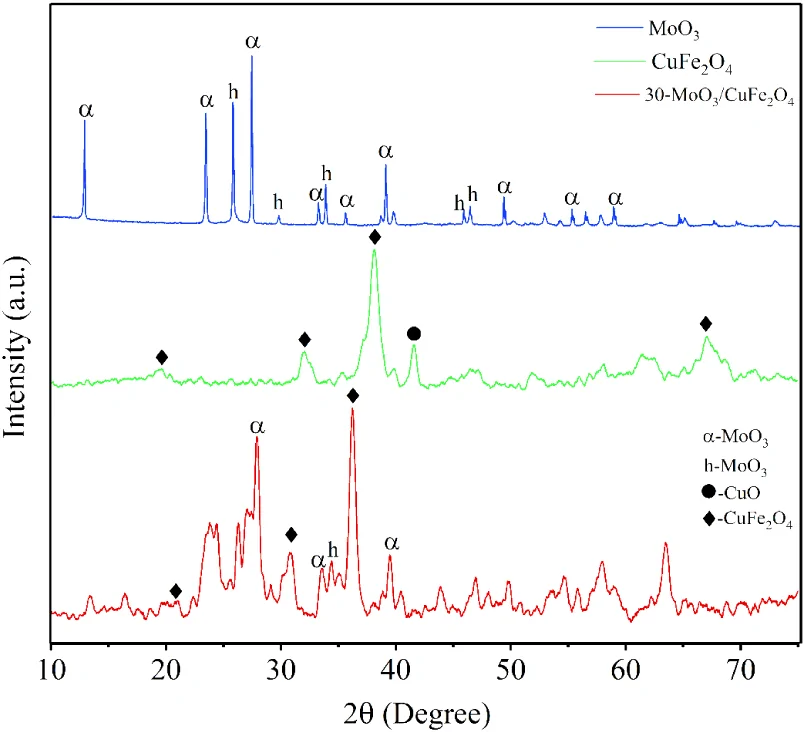
XRD patterns of MoO_3_, CuFe_2_O_4_, and the catalyst 30-MoO_3_/CuFe_2_O_4_.

The diffractogram corresponding to CuFe_2_O_4_ shows four distinct peaks at 18.9°, 30.2°, 35.8°, and 62.7°, which correspond to the crystallographic planes (111), (220), (311), and (440) according to the standard card (ICDD 01–077–0010), indicating the formation of the inverse spinel structure of CuFe_2_O_4_.^34,35^ Furthermore, the single peak corresponding to CuO at 39.1° corresponds to the (111) plane of the monoclinic system (ICDD 03–065–2309). CuO is an impurity that may be related to the oxidation of copper species (Cu^0^ or Cu^+^) during synthesis under atmospheric conditions.^22^ The X-ray diffractogram referring to the 30-MoO_3_/CuFe_2_O_4_ catalyst shows characteristic peaks of the MoO_3_ structure, in its hexagonal form, at 33.8° (220), as well as in its orthorhombic form, at 27.3° (021), 33.1° (101), and 38.8° (060). Evidence of the presence of characteristic CuFe_2_O_4_ structure peaks can also be observed at shifted positions (approximately 20.8°, 31.0°, and 36.2°), corresponding to the (111), (220), and (311) planes, respectively, indicating that both material species constitute the catalyst. This shift is attributed to lattice distortions caused by the impregnation of MoO_3_ into the ferrite structure, confirming the formation of a composite material.

Figure 4 presents the FTIR analysis of MoO_3_, CuFe_2_O_4_, and 30-MoO_3_/CuFe_2_O_4_. In the MoO_3_ spectrum (blue line), bands are observed at 442 cm^–1^, 811 cm^–1^, 839 cm^–1^, and 977 cm^–1^, characteristic of the Mo–O and O–Mo–O bond-stretching vibrations.^20,22,23^ Additionally, the spectrum of the magnetic CuFe_2_O_4_ support (green line) indicated a band at 534 cm^–1^, attributed to metal–oxygen vibrations. In this case, the M–O stretching may involve either copper or iron as the metal, related to the spinel phase of the ferrite.^23^

**Figure 4:**
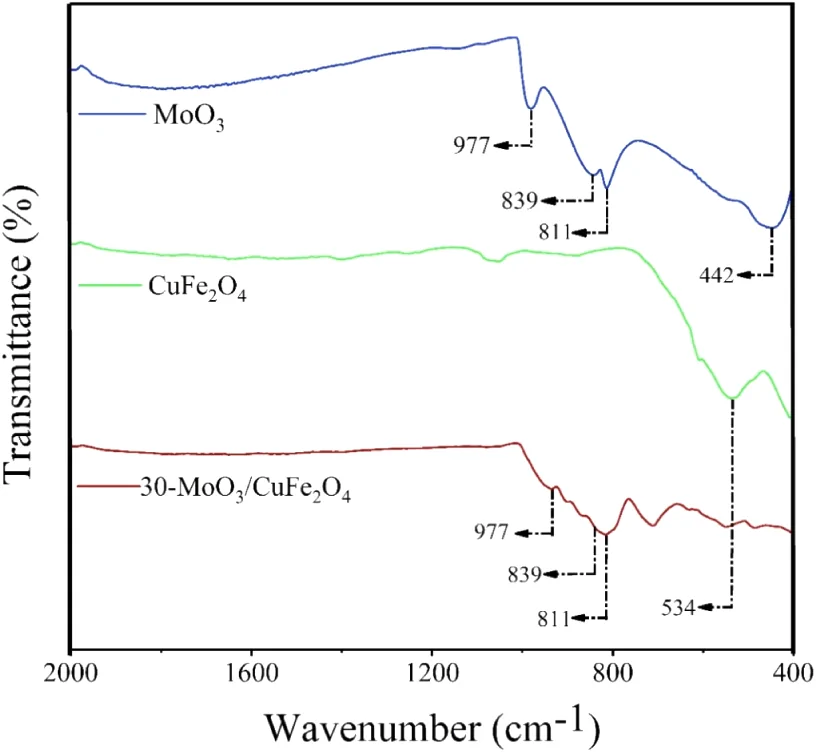
FTIR analysis of MoO_3_, CuFe_2_O_4_, and the 30-MoO_3_/CuFe_2_O_4_ catalyst.

Furthermore, in the spectrum of the 30-MoO_3_/CuFe_2_O_4_ catalyst (red line), a band at 534 cm^–1^ is observed, similar to that found in the spectrum of the CuFe_2_O_4_ support. Additionally, the bands identified at 811 cm^–1^, 839 cm^–1^, and 977 cm^–1^ demonstrate the presence of MoO_3_ in the catalyst, which indicates the presence of both structures (MoO_3_ and CuFe_2_O_4_) in the catalyst composition.

The morphologies of the MoO_3_, CuFe_2_O_4_, and 30-MoO_3_/CuFe_2_O_4_ materials are shown in Figure 5. Upon examining Figure 5(a), (b), and (c), the micrographs of the MoO_3_ structures exhibited rod-like agglomerates of different sizes, which are characteristic of the orthorhombic structure of α-MoO_3_. This structure is predominant in the synthesis of MoO_3_ at temperatures above 400 °C, as evidenced in the study by Brito et al.^26^

**Figure 5:**
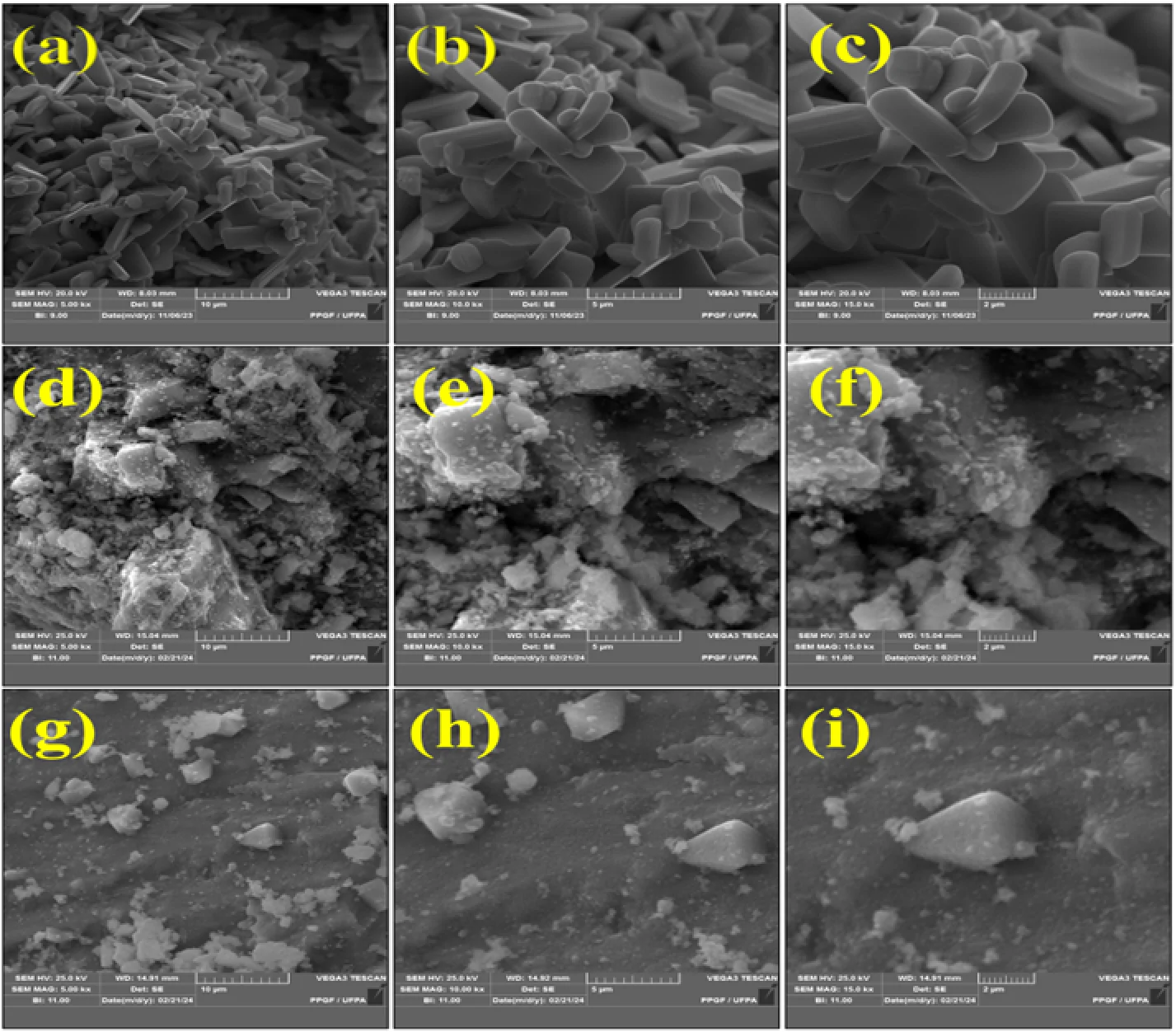
SEM images of (a) MoO_3_ 5 kx magnification, (b) MoO_3_ 10 kx magnification, (c) MoO_3_ 15 kx magnification, (d) CuFe_2_O_4_ 5 kx magnification, (e) CuFe_2_O_4_ 10 kx magnification and (f) CuFe_2_O_4_ 15 kx magnification, (g) 30-MoO_3_/CuFe_2_O_4_ 5 kx magnification, (h) 30-MoO_3_/CuFe_2_O_4_ 10 kx magnification, and (i) 30-MoO_3_/CuFe_2_O_4_ 15 kx magnification.

The images captured in the SEM micrographs corresponding to CuFe_2_O_4_ (Figure 5(d), (e), and (f)) reveal a rough and spongy appearance in the analyzed region. It is worth noting that this structure is similar to that developed in the study by Rajput et al.^36^ on the synthesis of a nanocatalyst supported on CuFe_2_O_4_. Thus, it is possible to observe structures formed by agglomerates of particles with a spongy appearance, with an estimated size in the range of 1.0 to 5.0 μm.^37^

In Figure 5(g), (h), and (i), the micrographs of the 30-MoO_3_/CuFe_2_O_4_ catalyst demonstrate the rough structure of the CuFe_2_O_4_ support surrounded by parts of the MoO_3_ active phase. Although the rod-like shapes in the figures do not exhibit the characteristic morphology shown in Figure 5(a), (b), and (c), the disappearance of the spongy aspect of the support is observed, which may be related to the dispersion of the MoO_3_ active phase onto the surface.

(Figure S1 Supporting Information) presents the results obtained by EDS composition analysis and surface elemental mapping of the CuFe_2_O_4_ support. The EDS spectrum of CuFe_2_O_4_ in (Figure S1a) displays the main peaks corresponding to the elements iron (Fe), copper (Cu), and oxygen (O) on the magnetic support surface, with concentrations of 49.4%, 28.2%, and 22.5%, respectively. Thus, the Fe/Cu ratio value is approximately 2:1, which is close to the target ratio intended during the synthesis step of this material and indicates the efficiency of the synthesis methodology. Furthermore, CuFe_2_O_4_ exhibited uniformly distributed elements on the support surface, as shown by the surface elemental mapping in (Figure S1b), indicating the effectiveness of the coprecipitation synthesis process employed.

The composition and surface elemental mapping determined by EDS for the components of the 30-MoO_3_/CuFe_2_O_4_ catalyst are shown in Figure 6. The elemental composition of the synthesized catalyst is shown in Figure 6(a). A molybdenum content of 32.1 wt % was determined, which closely matches the nominal loading of 30 wt % established during the synthesis procedure, indicating the successful incorporation of the active phase by the wet impregnation method. Figure 6(b) displays the elemental mapping of the catalyst along with the corresponding individual elemental maps, confirming the distribution of the constituent elements across the catalyst surface. Although small dark patches are observed in the oxygen and molybdenum maps, indicating local concentration variations, a common feature for supported catalysts prepared by impregnation, the overall distribution of the elements can be considered relatively homogeneous, according to their respective percentages. This local heterogeneity does not impair the catalytic performance, as demonstrated by the high conversion levels achieved.

**Figure 6:**
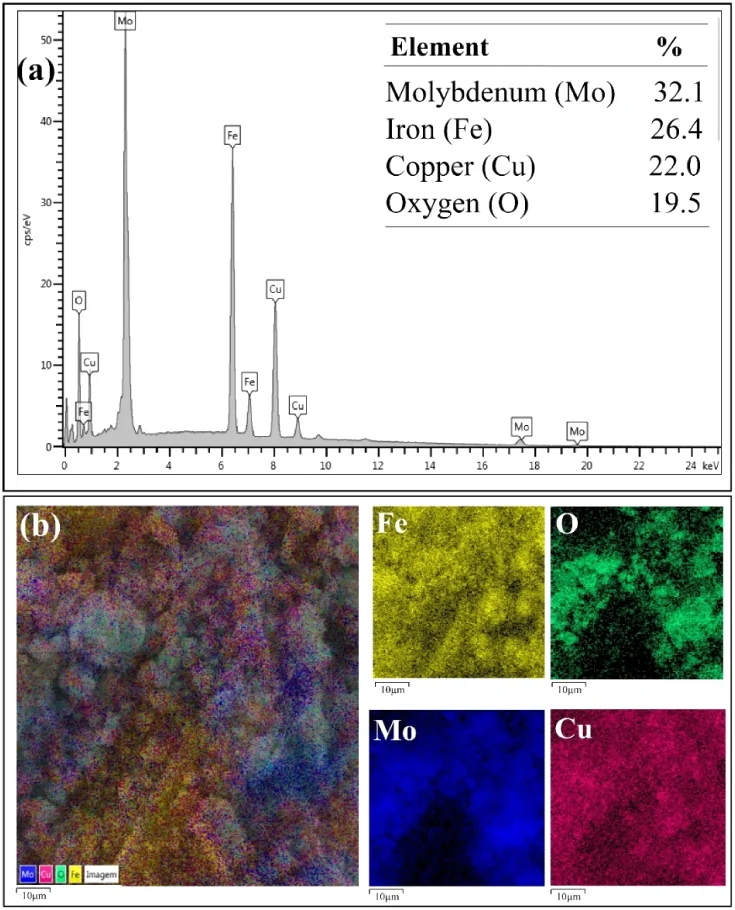
EDS results for the 30-MoO_3_/CuFe_2_O_4_ catalyst: (a) elemental composition and (b) elemental mapping showing the distribution of each constituent element.

The magnetic behavior of the CuFe_2_O_4_ support and the 30-MoO_3_/CuFe_2_O_4_ catalyst was investigated by vibrating sample magnetometry (VSM). The corresponding magnetization versus applied magnetic field (M–H) curves are presented in Figure 7.

**Figure 7:**
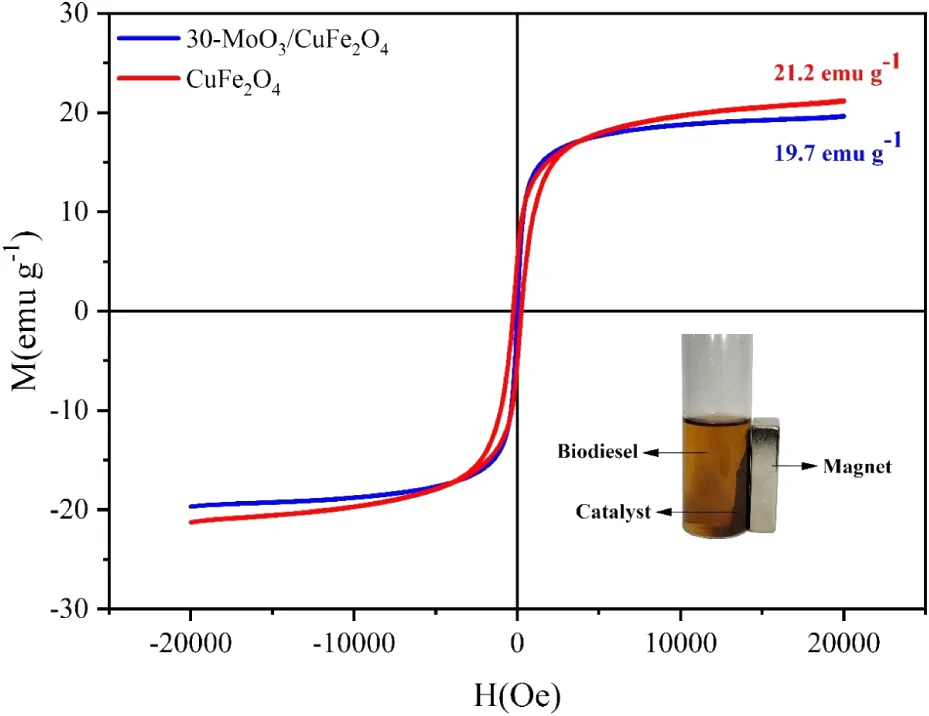
Magnetic hysteresis loops of CuFe_2_O_4_ and the catalyst 30-MoO_3_/CuFe_2_O_4_.

From the analysis of Figure 7, it is possible to observe the ferromagnetic behavior of both CuFe_2_O_4_ and 30-MoO_3_/CuFe_2_O_4_, with their saturation magnetization (Ms) values being 21.2 emu g^–1^ and 19.7 emu g^–1^, respectively. The Ms parameter indicates the maximum magnetization state achieved by the materials through the application of a magnetic field of ±20,000 Oe, demonstrating the strong magnetic responsiveness of the synthesized materials.

The magnetic activity potential of CuFe_2_O_4_ after the catalyst preparation process showed a reduction of approximately 8% in the catalyst’s magnetic activity, as the value decreases from 21.2 emu g^–1^ to 19.7 emu g^–1^. This slight decrease is associated with the introduction of a nonmagnetic species, such as MoO_3_, at a loading of approximately 30%. Furthermore, the thermal treatment at 450 °C to which the catalyst was subjected for the formation of the active phase may have contributed to the partial misalignment of spins and, consequently, decreased the magnetic moment of the material.^11^

The Ms value obtained for 30-MoO_3_/CuFe_2_O_4_ was sufficient for its separation by applying a magnetic field and is also considerably higher compared to other magnetic catalytic solids applied to biodiesel production. Recent studies, such as that by Saleh et al.,^38^ who investigated the application of the TiFe_2_O_4_@SiO_2_–SO_3_H magnetic catalyst in biodiesel production, observed an Ms value of 5.51 emu g^–1^. Another study, developed by Sun et al.,^39^ obtained an Ms value of 7.30 emu g^–1^ for the magnetic solid Fe_3_O_4_@UiO-66-NH_2_. Additionally, Tamoradi et al.^25^ developed the MgO@GO-CuFe_2_O_4_ catalyst and reported an Ms value of 14.9 emu g^–1^. The higher saturation magnetization of 30-MoO_3_/CuFe_2_O_4_ can be attributed to the intrinsically high magnetic response of the copper ferrite support, the low content (30 wt %) of the nonmagnetic MoO_3_ phase, and the absence of thick nonmagnetic coatings. This evidences that the 30-MoO_3_/CuFe_2_O_4_ catalyst exhibits a higher Ms value compared to other catalytic magnetic materials reported in the literature, thus enabling easy separation from the reaction medium by an external magnetic field, as shown in Figure 7.

### 3.3 Optimization of Transesterification Reaction Parameters

#### 3.3.1 Regression Model Development and Evaluation of the Effects of Process Variables on Ester Content

The performance of the 30-MoO_3_/CuFe_2_O_4_ catalyst in biodiesel production was investigated by using a 2^4^ face-centered central composite design (FCCD). Thirty experimental runs were performed in a randomized order, and the corresponding ester content values of the biodiesel samples are summarized in Table 2.

**Table 2:** FCCD Matrix and Experimental Results for Biodiesel Production Using the 30-MoO_3_/CuFe_2_O_4_ Catalyst

		Independent variables				Response
Std. order	Run order	X_1_ (°C)	X_2_ (mol/mol)	X_3_ (wt %)	X_4_ (h)	Y_1_(%)
1	4	120	10	2	1	36.9
2	14	120	10	2	5	42.1
3	10	120	10	10	1	43.4
4	8	120	50	2	1	35.7
5	12	180	10	2	1	79.9
6	9	180	50	10	1	91.8
7	15	180	50	2	5	85.8
8	28	180	10	10	5	79.9
9	20	120	50	10	5	82.5
10	2	180	50	10	5	87.1
11	17	180	50	2	1	81.8
12	13	180	10	10	1	80.6
13	26	180	10	2	5	76.8
14	24	120	10	10	5	59.6
15	16	120	50	2	5	58.7
16	30	120	50	10	1	42.2
17	25	120	30	6	3	49.2
18	5	150	10	6	3	76.9
19	23	150	30	2	3	89.1
20	11	150	30	6	1	80.9
21	19	180	30	6	3	77.8
22	21	150	50	6	3	85.4
23	7	150	30	10	3	86.5
24	29	150	30	6	5	91.9
25	27	150	30	6	3	88.2
26	6	150	30	6	3	89.9
27	3	150	30	6	3	90.9
28	22	150	30	6	3	91.2
29	18	150	30	6	3	89.8
30	1	150	30	6	3	90.7

The results presented in Table 2 indicate that the ester content of the biodiesel varied from 36.9% (Experiment 1) to 91.9% (Experiment 24), demonstrating the strong influence of the investigated process variables on the transesterification reaction. The experimental data were subsequently used to develop a quadratic regression model to evaluate the effects of independent variables X_1_, X_2_, X_3_, and X_4_ on the ester content. The statistical significance and adequacy of the model were assessed by analysis of variance (ANOVA) at a 95% confidence level, as summarized in Table 3. Variables with *p* values lower than 0.05 were considered statistically significant.

**Table 3:** ANOVA of the Second-Order Model for Transesterification Optimization Using the 30-MoO_3_/CuFe_2_O_4_ Catalyst^a^

Source	Sum of squares	Degree of freedom	Mean squares	F-value	Prob. > F	Remarks
Model	9846.74	15	656.44	15.50	<0.0001	Significant
X_1_	4710.97	1	4710.969	3959.907	<0.0001	Significant
X_1_^2^	1203.22	1	1203.225	1011.396	<0.0001	Significant
X_2_	311.67	1	311.667	261.979	<0.0001	Significant
X_2_^2^	39.41	1	39.408	33.125	0.0222	Significant
X_3_	247.90	1	247.902	208.380	<0.0001	Significant
X_3_^2^	19.59	1	19.594	16.470	<0.0001	Significant
X_4_	462.08	1	462.080	388.411	<0.0001	Significant
X_4_^2^	4.72	1	4.722	3.969	0.1029	Not significant
X_1_X_2_	3.80	1	3.803	3.196	0.1338	Not significant
X_1_X_3_	96.04	1	96.040	80.728	<0.0001	Significant
X_1_X_4_	497.29	1	497.290	418.008	<0.0001	Significant
X_2_X_3_	11.90	1	11.903	10.005	0.0250	Significant
X_2_X_4_	126.56	1	126.563	106.385	<0.0001	Significant
X_3_X_4_	30.25	1	30.250	25.427	0.0039	Significant
Residual	351.64	15	-	-	-	
Lack of fit	345.69	10	34.569	29.058	0.0008	Significant
Pure error	5.95	5	1.19			
Total SS	10198.38	29				
R^2^ = 0.9655						
R^2^ Adj. = 0.9333						

a Statistically significant at the confidence level of 95.0% (α = 0.05).

Based on the analysis of Table 3, the effects of the independent variables temperature (X_1_ and X_1_^2^), MeOH:WCO molar ratio (X_2_ and X_2_^2^), catalyst concentration (X_3_ and X_3_^2^), and time (X_4_) were significant, as well as the interactions between these variables X_1_X_3_, X_1_X_4_, X_2_X_3_, X_2_X_4_, and X_3_X_4_. The only exceptions are the interaction between temperature and MeOH:WCO molar ratio (X_1_X_2_) and the quadratic effects of the independent variable reaction time (X_4_^2^), as they have *p* values greater than 0.05 (95% confidence level) and are therefore considered insignificant within the stipulated confidence interval.

Furthermore, Fisher’s F-test (eq 2) demonstrated the adequacy of the proposed regression model, as the calculated F-value (15.50) was considerably higher than the corresponding tabulated value (2.48). This result confirms that the model provides reliable predictions of ester content at the 95% confidence level. The final polynomial (eq 5) was then obtained by regression analysis after removing the statistically insignificant terms identified through ANOVA.

5 $$Y_{1}=87.58+32.35X_{1}-43.10X_{1}^{2}+8.32X_{2}-7.6X_{2}^{2}+7.42X_{3}+5.50X_{3}^{2}+10.13X_{4}-4.90X_{1}X_{3}-11.15X_{1}X_{4}+1.72X_{2}X_{3}+5.62X_{2}X_{4}+2.75X_{3}X_{4}$$

The predictive performance of eq 5 was further assessed by comparing the predicted and experimental ester content values and by examining the corresponding residuals. Figure 8 presents the plots of predicted versus experimental values, the residual distribution, residuals as a function of the predicted values, and the Pareto chart of the standardized effects, providing a comprehensive evaluation of the statistical model.

**Figure 8:**
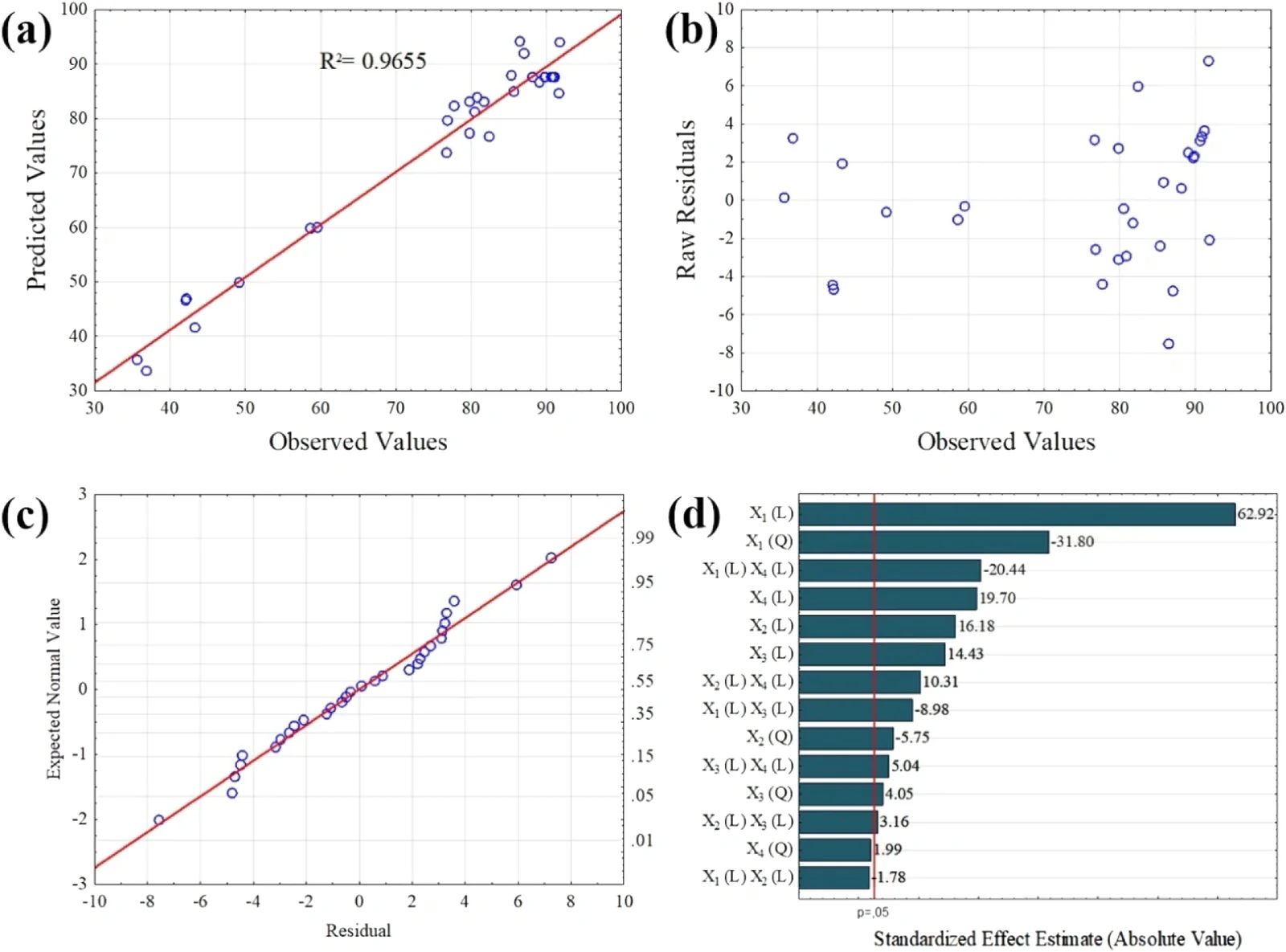
Plots of (a) predicted vs experimental ester content, (b) raw residuals vs predicted values, (c) normal probability plot of residuals, and (d) Pareto chart of standardized effects and *p* values for ester content.

In this respect, Figure 8(a) shows the correlation plot of predicted versus observed values and, therefore, confirms a high correlation between these values, since the coefficient of determination R^2^ and adjusted R^2^ values are 0.9655 and 0.9333, respectively. Therefore, this result exhibits a high correlation between predicted and observed values (R^2^ = 0.9655), indicating that the developed model can explain approximately 97% of the Y_1_ values obtained within the studied range of variables. These results demonstrated the adequacy of the model to the data, as well as expressing high accuracy in predicting ester content within the studied levels.^20,40^

Figure 8(b) and 8(c) presents the residuals versus predicted values and the normal probability plot of the residuals, respectively. As shown in Figure 8(b), the raw residuals, calculated as the difference between the experimental and predicted ester content values, are randomly distributed around zero. This random pattern indicates that the assumptions of independence and homoscedasticity (constant variance) are satisfied, supporting the adequacy of the proposed quadratic regression model.^41^ In addition, the normal probability plot shown in Figure 8(c) exhibits an approximately linear distribution of the residuals, indicating that the errors follow a normal distribution and do not significantly affect the validity of the fitted model.^11,12^

Figure 8(d) displays the Pareto diagram of the developed study, indicating the significance level of the studied variables. The significant terms of the model are represented by the values that exceed the vertical red dashed line (*p* value = 0.05). According to the Pareto analysis, reaction temperature was identified as the most significant factor influencing the transesterification process. Both the linear (X_1_) and quadratic (X_1_^2^) effects were statistically significant, exhibiting standardized effects of 62.92 and −31.80, respectively. Therefore, these values indicated that increasing the reaction temperature favors the increase in ester conversion in the transesterification reaction.^19^ However, the negative influence of the quadratic variable (X_1_^2^), observed in Figure 8(d), indicated that increasing temperature favors ester conversion up to a certain range within the studied interval, beyond which it becomes detrimental to biodiesel synthesis, decreasing the conversion rate.^19,20,23^

Regarding the MeOH:WCO molar ratio variable, both its linear effect (X_2_) and quadratic effect (X_2_^2^), with values of 16.18 and −5.75, respectively, indicated that increasing the X_2_ value promotes an increase in ester conversion during the transesterification reaction process. Thus, the analysis of the quadratic variable effect (X_2_^2^) in the reaction medium is pertinent, similar to X_1_^2^, since biodiesel synthesis would be significantly impaired by an excessive amount of MeOH:WCO in the reaction medium.^20^

Furthermore, it was observed that for catalyst concentration, both its linear effect (X_3_) (14.43) and its quadratic effect (X_3_^2^) (4.05) demonstrated that increasing the catalyst concentration resulted in an increase in the ester content value of the response variable.^23,27^ Considering the reaction time variable in its linear effects (X_4_), with a value of 19.70, it indicated its significance and that increasing the reaction time increased the conversion of the reaction process, while its quadratic effects (X_4_^2^) (1.99) became insignificant.^27^

The interactions of temperature with the MeOH:WCO molar ratio (X_1_X_2_) proved to be insignificant (−1.78), as observed in Figure 8(d). The interaction between these variables presented negative values, and consequently, the maximization of one variable occurred at the expense of the other, making the system unfavorable, both by the maximization of the X_1_ effect and the X_2_ effect. This is because excessively increasing temperature becomes detrimental to the transesterification reaction and methyl ester formation, as well as leading to excess methanol in the gas phase, which can reduce its availability in the reaction medium, impairing the ester conversion content in biodiesel.^12,18^ The interactions between temperature and catalyst concentration (X_1_X_3_) and reaction time (X_1_X_4_) proved to be negative with values of −8.98 and −20.44, highlighting the maximization of only one of the variables to the detriment of the other.^18^ The interactions between the molar ratio and catalyst concentration (X_2_X_3_) (3.16) and reaction time (X_2_X_4_) (10.31) are significant; thus, their interactions benefited from the maximization of both variable values.^19^ Finally, the interaction between catalyst concentration and reaction time (X_3_X_4_) (5.04) demonstrated a positive and significant value.

Response surface methodology (RSM) was applied to investigate the interactive effects of the independent variables (X_1_, X_2_, X_3_, and X_4_) on ester content (Y_1_) and to identify the optimal operating conditions for the transesterification process. Six three-dimensional response surface plots were generated, each describing the combined influence of two variables, while the remaining variables were fixed at their central levels. The response surfaces are shown in Figure 9.

**Figure 9:**
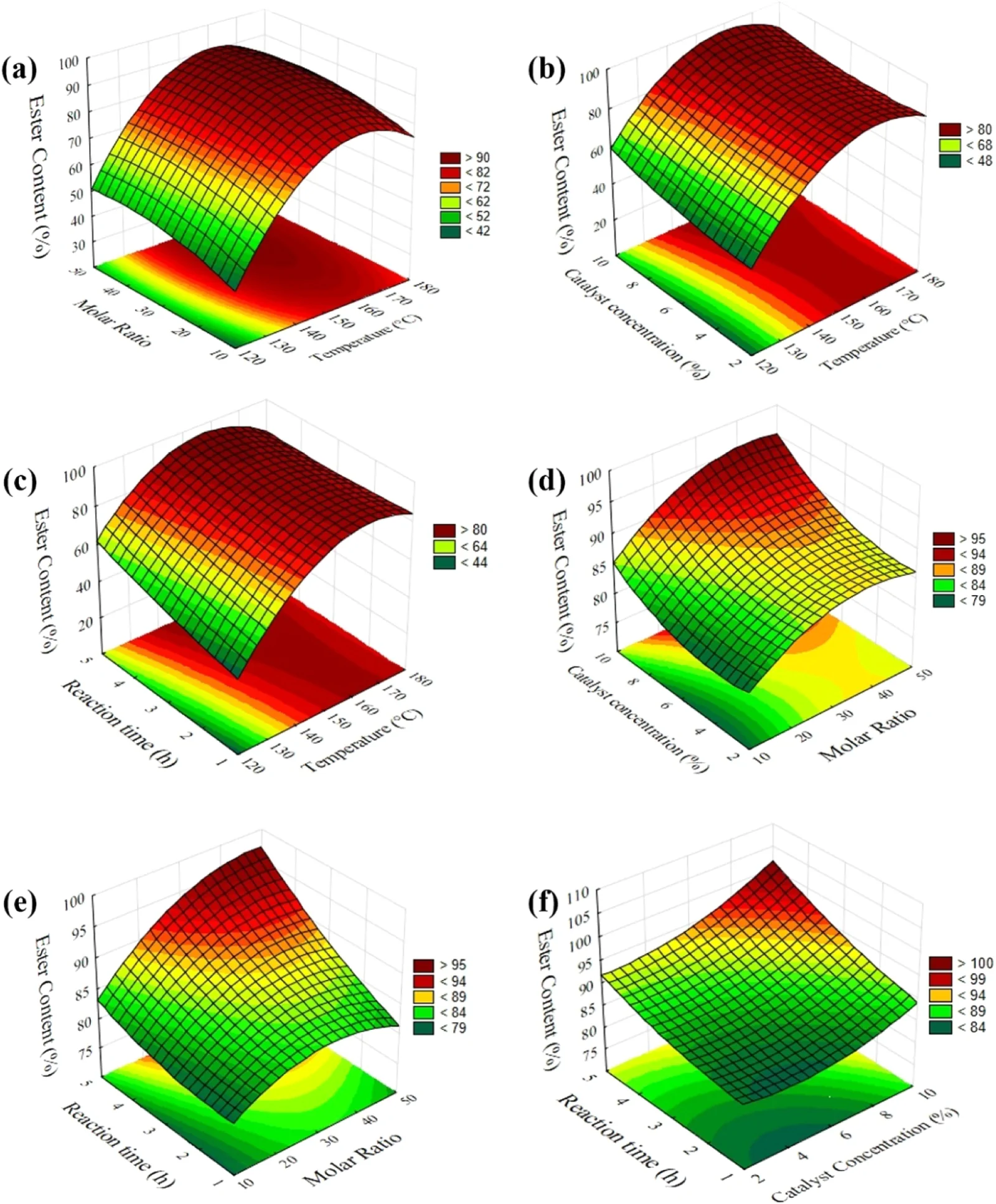
Surface plots illustrating the combined effects of (a) reaction temperature and molar ratio, (b) catalyst concentration and reaction temperature, (c) reaction temperature and reaction time, (d) molar ratio and catalyst concentration, (e) molar ratio and reaction time, and (f) catalyst concentration and reaction time on biodiesel ester content.

Figure 9(a)–(c) presents the response surface and contour plots describing the interactions between reaction temperature (X_1_) and the methanol-to-oil molar ratio (X_2_), catalyst concentration (X_3_), and reaction time (X_4_), respectively. Analysis of these surfaces indicates that the ester content (Y_1_) increases with increasing reaction temperature. This behavior can be attributed to the enhanced molecular collisions, improved phase miscibility, and greater mass transfer promoted by higher temperatures, which favor the transesterification reaction and consequently increase biodiesel ester content.^21–23,41^ This trend is consistent with the Pareto analysis, which identified significant positive linear and quadratic effects associated with the temperature variable. However, at temperatures above approximately 170 °C, a decline in ester content is observed in all three response surfaces, indicating that excessively high temperatures adversely affect the process. This behavior reflects the negative quadratic effect of temperature (X_1_^2^) and is further evidenced by its interaction with the other process variables, particularly the X_1_X_2_ interaction, followed by X_1_X_3_ and X_1_X_4_, as indicated in the Pareto chart.^42^

Figure 9(d) and (e) presents the response surface and contour plots illustrating the interactions between the molar ratio (X_2_) and catalyst concentration (X_3_) and between the molar ratio (X_2_) and reaction time (X_4_), respectively. As shown in Figure 9(d), low values of both the molar ratio and catalyst concentration resulted in ester contents below 80%, indicating that these variables exert a synergistic effect on the transesterification process. An increase in both X_2_ and X_3_ led to higher ester contents (Y_1_). This behavior can be attributed to the higher availability of methanol, which promotes the formation of methoxy species on the catalyst surface and enhances their interaction with acidic active sites. At the same time, increasing the catalyst concentration provides a greater number of active sites, improving mass transfer and facilitating the conversion of triglycerides into methyl esters.^26–28^

Figure 9(e) presents the interaction between the molar ratio and time. It is possible to observe ester contents above 90% only at molar ratios above 30:1, with time above 3 h, showing that variable X_2_ has a strong interaction with variable X_4_ when both are at high values and above the averages applied in the study. This increase in the ester content due to the increase in time confirms the analysis performed in the Pareto diagram, in which variable X_4_ has a significant positive linear value. Furthermore, an increase in the contact time between the catalyst and reactants promotes a greater number of collisions, making the conversion of triglycerides into biodiesel more favorable.^30^

Finally, Figure 9(f) presents the response surface and contour plots corresponding to the interaction between the catalyst concentration (X_3_) and reaction time (X_4_). As observed, increasing both variables results in a higher ester content (Y_1_), indicating that a greater number of active sites combined with a longer reaction time enhances the conversion of triglycerides into methyl esters. Thus, it is possible to observe that, due to their positive interaction, the two variables interact more favorably when their values are maximized, increasing the ester content to above 94%, only at the intersection of X_3_X_4_ at extreme values in the surface plot, between 5 h of reaction time and 10% catalyst concentration.^19,20^

#### 3.3.2 Optimization and Validation of the Developed Model

To determine the optimal operating conditions for biodiesel production, the transesterification process variables (X_1_, X_2_, X_3_, and X_4_) were optimized using Derringer’s desirability function implemented in TIBCO Statistica software (version 14). This multiresponse optimization approach identified the combination of process parameters that maximized the predicted ester content. The optimum reaction conditions were found to be a reaction temperature of 155 °C, a MeOH:WCO molar ratio of 35:1, a catalyst loading of 8.0 wt %, and a reaction time of 3.5 h. Under these conditions, the regression model predicted an ester content of 94.4%, as illustrated in Figure 10.

**Figure 10:**
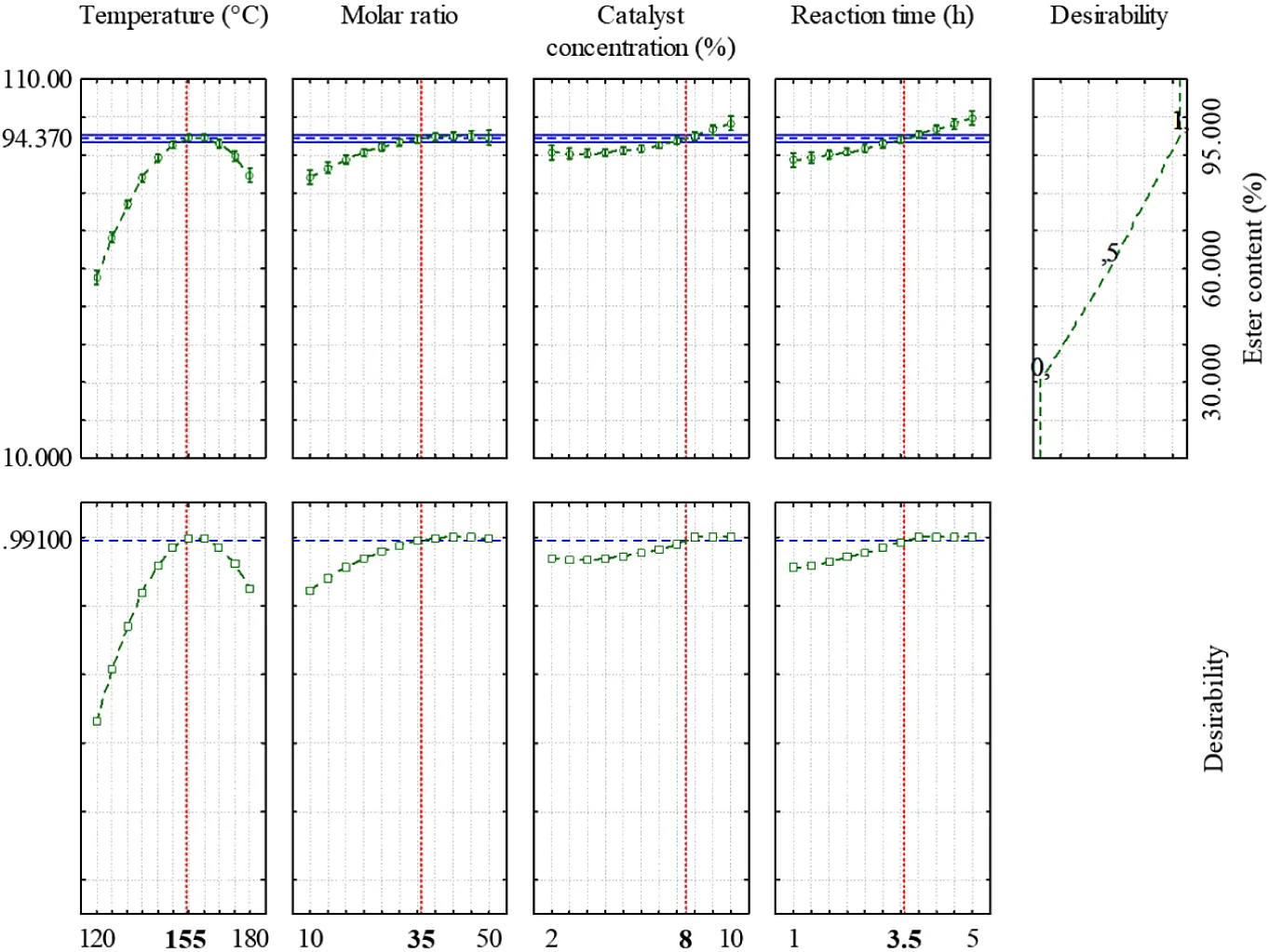
Optimum operating conditions predicted by the regression model for biodiesel production catalyzed by the 30-MoO_3_/CuFe_2_O_4_ catalyst.

The reliability of the developed model and the independent variables selected for the process was evaluated and validated through the execution of three experiments. The optimized values obtained from the regression model were used in experiment 1, while experiments 2 and 3 were carried out under random conditions in order to study the predictive capacity of the developed model for different reaction conditions within the studied range. (Table S1 Supporting Information) shows the experimental and predicted ester content values, as well as the relative errors.

To validate the regression model, transesterification experiments were conducted under optimized reaction conditions. The experimental ester content reached 95.2%, corresponding to a relative error of only 0.84% with respect to the predicted value (94.4%), thereby demonstrating the excellent predictive accuracy of the proposed model. Additional validation experiments (experiments 2 and 3) yielded ester contents of 74.2% and 54.4%, with relative errors of 1.08% and 4.39%, respectively, further confirming the reliability of the regression model over a broader range of operating conditions. Thus, the data demonstrate the validity of the correlations as well as the reliability of the experimental procedure, in addition to showing excellent predictive capacity of the regression model developed in this study.

### 3.4 Characterization of the Synthesized Biodiesel

The quality of the biodiesel produced under the optimized transesterification conditions using the 30-MoO_3_/CuFe_2_O_4_ catalyst (reaction temperature of 155 °C, MeOH:WCO molar ratio of 35:1, catalyst loading of 8 wt %, and reaction time of 3.5 h) was assessed through the determination of its physicochemical properties, as summarized in Table 4. The analyzed parameters included density at 20 °C, kinematic viscosity at 40 °C, copper strip corrosion, acid value, flash point, and cold filter plugging point (CFPP). The experimental results were evaluated according to the specifications established by the ASTM D6751 standard to verify the suitability of the produced biodiesel as a transportation fuel.

**Table 4:** Physicochemical Properties of Biodiesel Produced by the 30-MoO_3_/CuFe_2_O_4_ Magnetic Catalyst and Their Limits

Test description	Unit	Test method	ASTM D6751 limits	Present study
Kinematic viscosity (at 40 °C)	mm^2^ s^–1^	D445	1.90–6.00	4.34
Density (at 20 °C)	g cm^–3^	D6890	0.875–0.900	0.889
Acid value	mg KOH g^–1^	D664	0.50 max	0.22
Flash point	°C	D93	130.0 min	168.0
Cold filter plugging point	°C	D6371	NS	1.0
Copper strip corrosion	-	D130	3 max	1a^a^

a ASTM D130 classification 1a: slight uniform tarnish (orange or yellowish). NS = not specified.

The density and kinematic viscosity of biodiesel are key properties influencing fuel injection performance and atomization, while also serving as indicators of impurities that may contribute to deposit formation in engine components.^12,22^ The biodiesel produced in this study exhibited a density of 0.889 g cm^–3^ and a kinematic viscosity of 4.34 mm^2^ s^–1^, both of which fall within the limits specified by international standards. As shown in Table 4, the copper strip corrosion and acid value were 1a and 0.22 mg KOH g^–1^, respectively.

These parameters are closely associated with the presence of free fatty acids and metallic contaminants, which can promote corrosion and compromise the durability of engine components. In addition, the flash point and cold filter plugging point (CFPP) were 168 and 1.0 °C, respectively. Overall, all physicochemical properties evaluated for the biodiesel produced in this study complied with the requirements established by the applicable international standards.^12,22,27^

### 3.5 Reusability Study of the 30-MoO_3_/CuFe_2_O_4_ Catalyst

The catalyst was used for four cycles in order to test its stability and recovery process. Thus, Figure 11 shows the ester content after each cycle of the 30-MoO_3_/CuFe_2_O_4_ catalyst, all carried out under optimized reaction conditions: reaction temperature of 155 °C, MeOH:WCO molar ratio of 35:1, catalyst concentration of 8 wt %, and reaction time of 3.5 h. Each reuse cycle was performed once. The experimental error, based on triplicate validation runs (Table S1), was below 5%.

**Figure 11:**
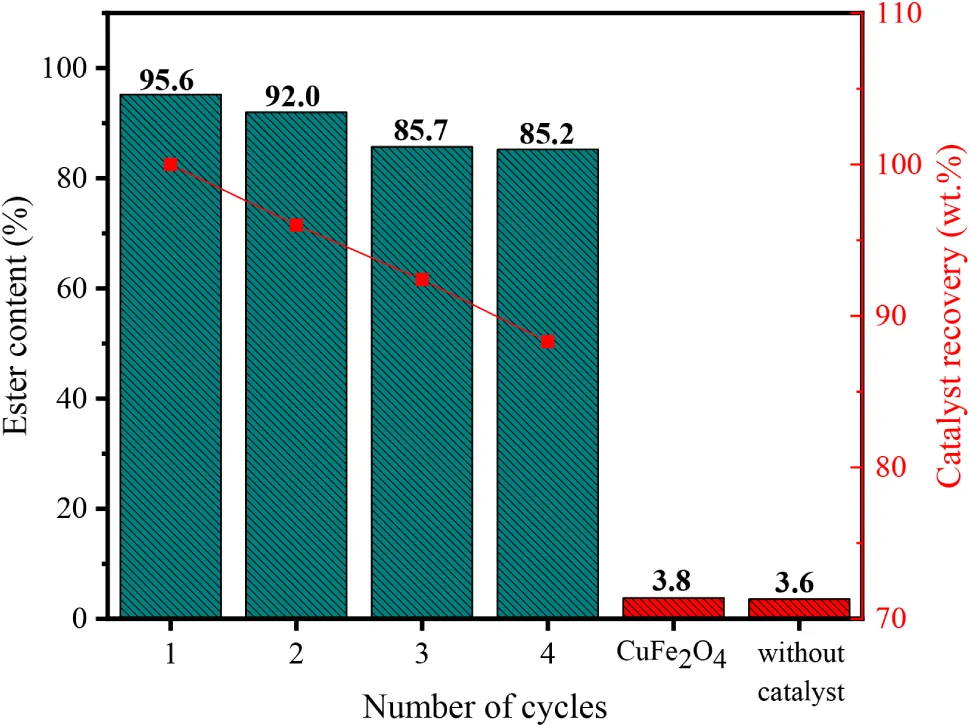
Reusability results of the catalyst 30-MoO_3_/CuFe_2_O_4_.

The first and second cycles presented ester content values of 95.6% and 92.0%, respectively. Thereafter, a decrease in methyl ester conversion is noticeable from the third cycle (85.7%), with a reduction of 9.9% compared to the first reaction cycle (95.6%). However, a relatively low decrease occurred in the fourth cycle (85.2%) compared to the third cycle. The decrease in catalytic activity may be due to leaching of the active phase material from the catalyst during the transesterification reaction, which explains the reduction in the surface acidity value determined after the last reuse, from 8.42 to 5.69 mmol H^+^ g^–1^.^18,19^ ^,^

The mass recovery of the catalyst was also monitored throughout the cycles. The recovered masses corresponded to 96% (cycle 2), 92.4% (cycle 3), and 88.3% (cycle 4) of the initial mass (0.96 g). The observed losses are mainly due to the washing, transfer, and drying steps of the catalyst and not to the magnetic separation, which proved adequate to retain the solid in the reaction medium. The cumulative loss of approximately 11.7% after four cycles is considered to be acceptable for reuse processes involving catalyst handling. This small mass loss, together with the reduction in surface acidity, contributes to the gradual decline in catalytic activity but does not compromise the good stability of the material since ester conversion remained above 85% in all cycles.

### 3.6 Comparison of Heterogeneous Acid Catalysts Reported for Biodiesel Production

Table 5 provides a comparative assessment of the catalytic performance of the 30-MoO_3_/CuFe_2_O_4_ catalyst relative to heterogeneous acid catalysts reported in the literature for biodiesel production, highlighting the effectiveness of the catalyst developed in the present study. The results demonstrate that the catalyst developed in this study achieved an ester yield comparable to those described in the literature while operating under relatively milder reaction conditions, namely, a reaction temperature of 155 °C, a MeOH:WCO molar ratio of 35:1, a catalyst loading of 8.0 wt %, and a reaction time of 3.5 h presents a lower reaction temperature, shorter reaction time, catalyst concentration and reduced methanol-to-oil molar ratio compared with many literature reports, resulted in comparable or even superior catalytic performance in several cases. These findings demonstrate the excellent catalytic activity of the 30-MoO_3_/CuFe_2_O_4_ catalyst and suggest that its use may enable a more economically viable biodiesel production process by reducing energy consumption and reagent requirements while maintaining high conversion efficiency.

**Table 5:** Comparison of Reaction Conditions and Catalytic Performance of Heterogeneous Acid Catalysts for Biodiesel Production^a^

		Operating conditions					
Catalysts	Triglyceride source	X_1_ (°C)	X_2_ (mol/mol)	X_3_ (wt %)	X_4_ (h)	Ester content (%)	References
MoO_3_/NiFe_2_O_4_	WCO	168	30:1	10.0	1.0	95.4	Gonçalves et al. (2024)^11^
WO_3_/CuFe_2_O_4_	WCO	180	45:1	6.0	3.0	95.2	Santos et al. (2022)^22^
MoO_3_/BaFe_2_O_4_	WCO	172	28:1	7.5	4.0	96.4	Gonçalves et al. (2026)^27^
MoO_3_/MnFe_2_O_4_	WCO	164.5	32:1	6.5	2.5	96.7	Santos et al. (2025)^28^
MoO_3_/GO	WCO	140	35:1	6.0	5.0	95.6	Silva et al. (2023)^43^
Mo/Naβ	Rice bran oil	147	19.4:1	1.5	8.0	84.6	Chen et al. (2020)^44^
MoAl@H-SiO_2_	Soybean oil	130	35:1	8.0	8.0	94.2	Li et al. (2024)^45^
Mo_15_W_15_/HSAPO-34/70	Soybean oil	140	16:1	14.5	48.0	90.1	Zhang et al. (2024)^46^
5Mo/ZSM-22	WCO	140	9:1	2.0	12.0	78.2	Zhang et al. (2022)^47^
30-MoO_3_/CuFe_2_O_4_	WCO	155	35:1	8.0	3.5	95.6	**This study**

a Note: Methanol was used as the alcohol in all studies.

Furthermore, besides its catalytic activity, the magnetic catalyst developed in this study offers the advantage of fast and straightforward separation by simply applying an external magnetic field, which can be particularly convenient for catalyst recovery in large-scale or continuous processes. Therefore, this catalyst offers the advantage of a less costly separation of the reaction medium, and its performance is similar to or even superior to that of various other solid acidic catalysts, whether magnetic or not, reported in the literature.

From Table 5, it is observed that the catalysts differ markedly in their optimal reaction parameters. The MoO_3_/MnFe_2_O_4_ catalyst (row 3) shows a slightly higher ester content (96.7%) than the 30-MoO_3_/CuFe_2_O_4_ catalyst (95.6%), operating at a lower methanol:oil ratio (32:1 vs 35:1) and shorter reaction time (2.5 h vs 3.5 h), but at a higher temperature (164.5 °C vs 155 °C). Similarly, the MoO_3_/BaFe_2_O_4_ catalyst (row 2) achieves 96.4% ester content at a higher temperature (172 °C) and longer reaction time (4 h) yet with a much lower methanol:oil ratio (28:1). These differences highlight the influence of the ferrite support composition (Mn, Ba, Cu) and the intrinsic acidity of the catalysts on the optimal reaction conditions. The 30-MoO_3_/CuFe_2_O_4_ catalyst offers a balanced performance with 95.6% ester content under relatively mild conditions (155 °C, 35:1, 8 wt %, 3.5 h), in addition to the practical advantage of fast and straightforward magnetic separation.

It is important to note, however, that heterogeneous acid catalysts in general require a relatively high methanol-to-oil ratio, as well as longer reaction times and higher temperatures than other common catalytic systems, such as heterogeneous base catalysts.^12,13^ These factors may pose challenges for methanol recovery and process throughput in industrial scale-up. Future studies should therefore explore strategies to reduce the methanol-to-oil ratio and reaction time, for instance, by optimizing the catalyst composition, employing more efficient reactors (such as microreactors or reactive distillation), or integrating in situ methanol removal. Techno-economic assessments would also be valuable to evaluate the overall feasibility of the proposed magnetic catalyst for the commercial production of biodiesel.

## 4 Conclusion

This study demonstrated the potential of the novel magnetic acid catalyst 30-MoO_3_/CuFe_2_O_4_ for biodiesel production via the transesterification of waste cooking oil. Comprehensive characterization by surface acidity measurements, XRD, FTIR, SEM, EDS, and VSM confirmed the successful synthesis and desirable physicochemical properties of the catalyst. Under the optimized reaction conditions (reaction temperature of 155 °C, MeOH:WCO molar ratio of 35:1, catalyst loading of 8 wt %, and reaction time of 3.5 h), the catalyst achieved an ester content of 95.6%. The quadratic regression model exhibited excellent predictive performance (R^2^ = 0.9655), with deviations between predicted and experimental values below 5%, confirming its suitability for describing and optimizing the transesterification process. Furthermore, the magnetic catalyst retained its catalytic activity over four consecutive reaction cycles, maintaining ester contents above 85%, thereby demonstrating good reusability and operational stability. These findings highlight the 30-MoO_3_/CuFe_2_O_4_ catalyst as a promising and efficient material for the sustainable production of biodiesel from low-cost feedstocks.

## Supplementary Material



## Notes

EDS composition and elemental maps of CuFe_2_O_4_ (Figure S1) and validation results including comparison between data achieved by the predictive model and experimental runs (Table S1) (PDF)

## References

[1] Alrudayni A. M., Alnuaimi M. T., Amshawee A. M., Ali R., Thanon K. S., Alassadi N. M. (2026). Magnetic nanoparticles as recyclable catalysts in biodiesel
synthesis from microalgae. Sustainable Chem..

[2] Shohel M. A., Islam M. A., Siam M. T. R. (2026). Evaluation of
activation, performance
and reusability between eggshell-derived heterogenous and homogenous
catalysts for neem oil biodiesel. Chem. Eng.
J. Adv..

[3] Xie Y., Zhao J., Wang H., Wang S., Zhang Y., Li R. (2026). Research progress on
biodiesel production utilizing waste oils and
biomass-derived catalysts. Fuel Process. Technol..

[4] Ribeiro T. S., Sobrinho I. A., Silva A. C. D., Gonçalves M. A., Fonseca D. C., Barreto I. A. R., Costa M. L., Filho G. N. R., Conceição L. R. V. (2026). Valorization
of Amazonian murici
seed waste into a sulfonated biochar catalyst: Process optimization
and kinetic studies for sustainable biodiesel production. Chem. Eng. J.: green Sustainable.

[5] Kumar A., Bhayana S., Singh P. K. T., Tripathi A. D. V., Paul V., Balodi V., Agarwal A. (2025). Valorization of used cooking oil:
challenges, current developments, life cycle assessment and future
prospects. Discovery Sustain..

[6] Burmana A. D., Manurung R. I. T. (2026). Investigate biodiesel products using
heterogeneous
catalysts in variations lauric acid feedstock through elemental analysis
and emissions testing using diesel engines. Catal. Today.

[7] Mondal S., Gurumoorthy A. V. P. M., Moorthy I. (2026). Sustainable production of biodiesel
from non-edible kernel-derived feedstocks: A comprehensive review
of production processes, properties, and optimization. Results Chem..

[8] Ferreira L., Da Silva César A., Conejero M. A., César
da Silva Guabiroba R. (2018). A Voluntary Delivery Point in Reverse Supply Chain
for Waste Cooking Oil: An Action Plan for Participation of a Public-School
in the State of Rio de Janeiro, Brazil. Recycling.

[9] Lima R. P., da Luz da Luz P. T. S., Braga M., dos Santos
Batista P. R., da Costa C. E. F., Zamian J. R., Do Nascimento L. A. S., Rocha Filho G. N. (2017). Murumuru (*Astrocaryum murumuru* Mart.) butter and oils of buriti (*Mauritia flexuosa* Mart.) and pracaxi (*Pentaclethra macroloba* (Willd.)
Kuntze) can be used for biodiesel production: Physico-chemical properties
and thermal and kinetic studies. Ind. Crop.
Prod..

[10] Gonçalves, M. A. ; Santos, H. C. L. ; Mares, E. K. L. ; Conceição, L. R. V. Chapter 5: Industry Waste-based Feedstocks. In Developments In Biodiesel: feedstock, Production, And Properties, 1nd ed.; Balakrishna, R. G. ; Mohan, S. ; Sharara, T. Z. ; Royal Society of Chemistry, 2024.

[11] Gonçalves M. A., Santos H. C. L., Silva M. A. R., Viegas A. C., Filho G. N. R., Conceição L. R. V. (2024). Biodiesel production
from waste cooking
oil using an innovative magnetic solid acid catalyst based on Ni–Fe
ferrite: RSM-BBD optimization approach. J. Ind.
And Eng. Chem..

[12] Mares E. K. L., Gonçalves M. A., da Luz P. T. S., Rocha
Filho G. N., Zamian J. R., Conceição L. R. V. (2021). Acai
seed ash as a novel basic heterogeneous catalyst for biodiesel synthesis:
Optimization of the biodiesel production process. Fuel.

[13] Ribeiro T. S., Sobrinho I. A., Gonçalves M. A., Lima V. S., Figueira B. A. M., Filho G. N. R., Conceição L. R. V. (2024). Green
synthesis
of biodiesel from magnetic basic biochar derived from Amazonian murici
residual biomass: Optimization, kinetic, thermodynamic, and environmental
studies. J. Environ. Chem. Eng..

[14] Bastos R. R. C., Corrêa A. P. L., da Luz P. T. S., Rocha
Filho G. N., Zamian J. R., Conceição L. R. V. (2020). Optimization
of biodiesel production using sulfonated carbon-based catalyst from
an amazon agro-industrial waste. Energy Convers.
Manage..

[15] Corrêa A. P. L., Bastos R. R. C., Rocha
Filho G. N., Zamian J. R., Conceição L. R. V. (2020). Preparation
of sulfonated carbon-based catalysts from murumuru kernel shell and
their performance in the esterification reaction. RSC Adv..

[16] Correa A. P. L., Silva P. M. L., Gonçalves M. A., Bastos R. R. C., Filho G. N. R., Conceição L. R. V. (2023). Study
of the activity and stability
of sulfonated carbon catalyst from agroindustrial waste in biodiesel
production: Influence of pyrolysis temperature on functionalization. Arabian J. Chem..

[17] Adhithan B., Sachdeva G. (2025). Optimization and evaluation
of Fe and Zn-coated zeolite
catalysts for biodiesel engine emission reduction and performance
enhancement. Biomass Bioenergy.

[18] Gonçalves M. A., Santos H. C. L., Silva P. M. L., Correa A. P. L., Ribeiro T. S., Sobrinho I. A., Filho G. N. R., Conceição L. R. V. (2024). Catalytic
conversion of residual raw material into biodiesel using a superior
magnetic solid acid catalyst based on Zn–Fe ferrite: thermodynamic
and kinetic studies. RSC Adv..

[19] Gonçalves M. A., Mares E. K. L., da Luz P. T. S., Narciso da Rocha Filho G., Rafael Vieira da Conceição L., Conceição L. R. V. (2021). Statistical
optimization of biodiesel production from waste cooking oil using
magnetic acid heterogeneous catalyst MoO_3_/SrFe_2_O_4_. Fuel.

[20] Gonçalves M. A., Mares E. K. L., da Luz P. T. S., Zamian J. R., Rocha
Filho G. N., Castro H. F., Conceição L. R. V. (2021). Biodiesel
synthesis from waste cooking oil using heterogeneous acid catalyst:
Statistical optimization using linear regression model. J. Renew. Sustain. Energy.

[21] Gonçalves M. A., Santos H. C. L., Ribeiro T. S., Viegas A. C., Filho G. N. R., Conceição L. R. V. (2024). Boosting
biodiesel production of
waste frying oil using solid magnetic acid catalyst from agro-industrial
waste. Arabian J. Chem..

[22] Santos H. C. L., Gonçalves M. A., Viegas A. C., Figueira B. A. M., da Luz P. T. S., Rocha Filho G. N., Conceição L. R. V. (2022). Tungsten
oxide supported on copper ferrite: A novel magnetic acid heterogeneous
catalyst for biodiesel production from low quality feedstock. RSC Adv..

[23] Santos H. C. L., Gonçalves M. A., Lima V. S., Viegas A. C., Filho G. N. R., Conceição L. R. V. (2024). NaAlO_2_/CuFe_2_O_4_ as a novel basic magnetic heterogeneous
catalyst for effective biodiesel production: Synthesis, characterization,
and optimization via RSM-FCCD modeling approach. Process Saf. Environ. Prot..

[24] Xie W., Li J. (2023). Magnetic solid catalysts
for sustainable and cleaner biodiesel production:
A comprehensive review. Renewable Sustainable
Energy Rev..

[25] Tamoradi T., Kiasat A. R., Veisi H., Nobakht V., Besharati Z., Karmakar B. (2021). MgO doped magnetic
graphene derivative as a competent
heterogeneous catalyst producing biofuels via transesterification:
Process optimization through Response Surface Methodology (RSM). J. Env. Chem. Eng..

[26] Brito V. L., Gonçalves M. A., Santos H. C. L., Filho G. N. R., Conceição L. R. V. (2023). Biodiesel
production from waste frying oil using molybdenum over niobia as heterogeneous
acid catalyst: Process optimization and kinetics study. Renewable Energy.

[27] Gonçalves M. A., Santos H. C. L., Lima V. S., Silva H. J. F., Fonseca D. F., Ribeiro T. S., Silva B. S., Viegas A. C., Conceição L. R. V. (2026). A New
Magnetically Separable BaFe_2_O_4_ Acid Catalyst
for Sustainable Biodiesel Production: L_9_ Taguchi Optimization
and Robust Recyclability. ACS Omega.

[28] Santos H. C. L., Gonçalves M. A., Filho G. N. R., Conceição L. R. V. (2025). Environmentally
sustainable production of biodiesel from waste edible oil using Mn–Fe
supported magnetic acid heterogeneous catalyst: Optimization via Taguchi
L9 technique. Sustainable Chem. Pharm..

[29] Boehm H. P. (1994). Some aspects
of the surface chemistry of carbon blacks and other carbons. Carbon.

[30] Pinto B. F., Garcia M. A. S., Costa J. C. S., Moura C. R. V., Abreu W. C., Moura E. M. (2019). Effect of calcination temperature
on the application
of molybdenum trioxide acid catalyst: Screening of substrates for
biodiesel production. Fuel.

[31] Mohebbi S., Rostamizadeh M., Kahforoushan D. (2020). Effect of
molybdenum promoter on
performance of high silica MoO_3_/B-ZSM-5 nanocatalyst in
biodiesel production. Fuel.

[32] Figueiredo J. S. B., Souza N. A., Alves B. T. S., Silva A. M., Barbosa B. V. S., Huaman N. R. C., Menezes R. R., Alves J. J. N. (2025). MoO_3_ Catalyst Supported on Micro-Mesoporous Structure for Biodiesel
Production. Catal. Lett..

[33] Cardoso R. K. P., Silva G. V. A., Alves B. T. S., Freire V. A., Alves J. J. N., Barbosa B. V. S. (2022). Evaluation of the effect of Si/Mo
and oil/alcohol ratios in the production of biodiesel from soybean
oil. Arabian J. Chem..

[34] Zhao K., Fang X., Huang Z., Wei G., Zheng A., Zhao Z. (2021). Hydrogen-rich syngas production from
chemical looping gasification
of lignite by using NiFe_2_O_4_ and CuFe_2_O_4_ as oxygen carriers. Fuel.

[35] Mao N., Bai H., Geng C., Meng L., Li N. (2022). Insights into the micro-structures
and reactive behaviors of coal vitrinite and inertinite macerals with
CuFe_2_O_4_ in chemical looping combustion. Sustain. Energy Technol. Assess..

[36] Rajput J. K., Arora P., Kaur G., Kaur M. (2015). CuFe_2_O_4_ magnetic heterogeneous nanocatalyst: Low power sonochemical-coprecipitation
preparation and applications in synthesis of 4H-chromene-3-carbonitrile
scaffolds. Ultrason. Sonochem..

[37] Soufi A., Hajjaoui H., Elmoubarki R., Abdennouri M., Qourzal S., Barka N. (2022). Heterogeneous Fenton-like
degradation
of tartrazine using CuFe_2_O_4_ nanoparticles synthesized
by sol-gel combustion. Appl. Surf. Sci. Adv..

[38] Saleh M. Y., Obaid A., O S., Hussein Adhab S. M. A., H A. (2024). TiFe2O4@SiO2–SO3H: A novel
and effective catalyst
for esterification reaction. Heliyon.

[39] Sun X., Xu L., Yuan H., Wang G., Maleki B. (2024). Boosted conversion
of restaurant waste oil into biodiesel using Fe_3_O_4_@UiO-66-NH_2_ magnetic heterogeneous nanocatalyst and its
application on the diesel engine: Optimization via RSM. Renewable Energy.

[40] Conceição L. R. V., Carneiro L. M., Rivaldi J. D., De Castro H. F. (2016). Solid acid
as catalyst for biodiesel production via simultaneous esterification
and transesterification of macaw palm oil. Ind.
Crop. Prod..

[41] Conceição L. R. V., Carneiro L. M., Giordani D. S., Castro H. F. (2017). Synthesis of biodiesel
from macaw palm oil using mesoporous solid catalyst comprising 12-molybdophosphoric
acid and niobia. Renewable Energy.

[42] Yusuf B. O., Ganiyu S. A., Peedikakkal A. M. P., Oladepo S. A. (2026). ZnO/Cu-BTC metal-organic
frameworks as a heterogeneous catalyst for efficient transformation
of waste cooking oil into biodiesel. Biomass
Bioenergy.

[43] Silva P. M. L., Gonçalves M. A., Correa A. P. L., Luz P. T. S., Zamian J. R., Filho G. N. R., Conceição L. R. V. (2023). Preparation
and characterization of a novel efficient catalyst based on molybdenum
oxide supported over graphene oxide for biodiesel synthesis. Renewable Energy.

[44] Chen C., Cai L., Zhang L., Fu W., Hong Y., Gao X., Jiang Y., Li L., Yan X., Wu G. (2020). Transesterification
of rice bran oil to biodiesel using mesoporous NaBeta zeolite-supported
molybdenum catalyst: experimental and kinetic studies. Chem. Eng. J..

[45] Li K., Xie W. (2024). Enhanced biodiesel
production from low-value acidic oils using ordered
hierarchical macro–mesoporous MoAl@H-SiO_2_ catalyst. Fuel.

[46] Zhang G., Xie W. (2024). Hierarchical porous
SAPO-34 decorated with Mo and W oxides for concurrent
transesterification-esterifications for efficient biodiesel production
from acidic soybean oil. Renewable Energy.

[47] Zhang W., Wang C., Luo B., He P., Zhang L., Hu G. (2022). Efficient and economic transesterification
of waste cooking soybean
oil to biodiesel catalyzed by outer surface of ZSM-22 supported different
Mo catalyst. Biomass Bioenergy.

