# Supplementary material for: Sustainable Biodiesel Production over a Novel Magnetic Solid Acid Catalyst Derived from Copper Ferrite: Statistical Optimization and Catalytic Performance Evaluation
Source: ACS Omega. 2026 Sep 17;11(38):58126–40. doi: 10.1021/acsomega.6c08033 (PMC13628994; doi:10.1021/acsomega.6c08033)
Supplement: Supplementary file 1 [file ao6c08033_si_001.pdf]

Supporting Information

Sustainable biodiesel production over a novel magnetic solid acid catalyst derived from copper ferrite: Statistical optimization and catalytic performance evaluation

Gustavo Ferreira Leal[a], Matheus Arrais Gonçalves[a], Hiarla Cristina Lima dos Santos[a], Alexandre da Cas Viegas[b], Erica Karine Lourenço Mares[c], Leyvison Rafael Vieira da Conceição[a*]

[a] Federal University of Pará, Institute of Exact and Natural Sciences, Graduate in Chemistry Program, Laboratory of Catalysis and Oleochemical, 66075–110, Belém, Pará, Brazil.

[b] Federal University of Rio Grande do Sul, Institute of Physics, 90035–190, Porto Alegre, Rio Grande do Sul, Brazil.

[c] Federal Institute of Education, Science and Technology of Pará, Department of Education, Science and Teacher Education, 68629–020, Paragominas, Pará, Brazil.

*Corresponding author

E-mail address: rafaelvieira@ufpa.br (Conceição, L.R.V)

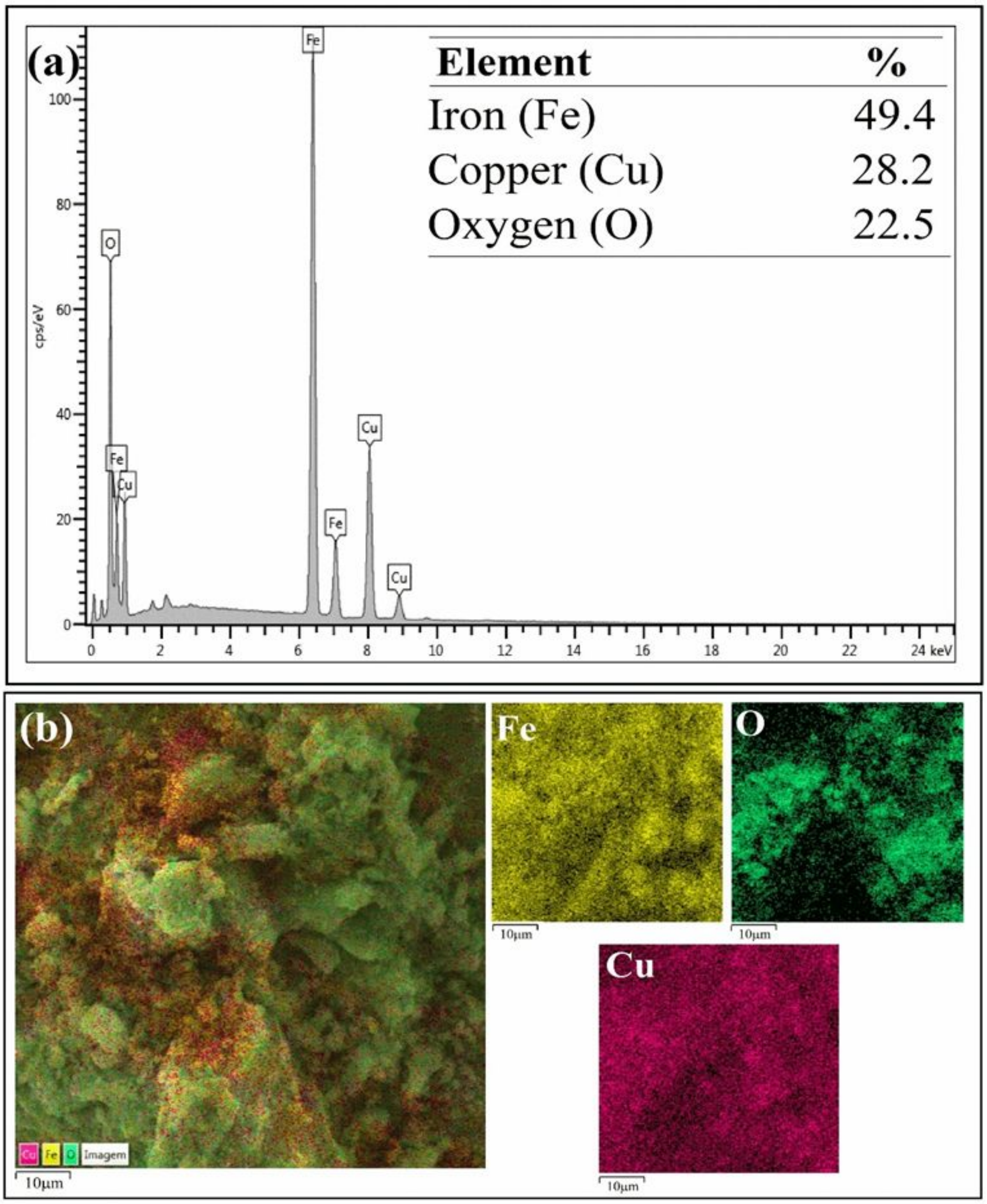

Figure S1. EDS composition of (a) $CuFe_2O_4$ and (b) elemental maps of each component of the $CuFe_2O_4$.

Table S1. Validation results: comparison between data achieved by predictive model and experimental runs.

Run	Transesterification conditions (X_1; X_2; X_3; X_4)	Predicted values (%)	Experimental values (%)	Relative error (%)
1 (OP)	155 ºC; 35:1; 8 wt.%; 3.5 h	94.4	95.2	0.84
2 (RC)	140 ºC; 24:1; 3 wt.%; 1.5 h	73.4	74.2	1.08
3 (RC)	130 ºC; 20:1; 4 wt.%. 1 h	56.9	54.4	4.39

OP: Optimal reaction condition; RC: Random reaction condition.
